# Transcriptomic and proteomic analyses of the *Aspergillus fumigatus *hypoxia response using an oxygen-controlled fermenter

**DOI:** 10.1186/1471-2164-13-62

**Published:** 2012-02-06

**Authors:** Bridget M Barker, Kristin Kroll, Martin Vödisch, Aurélien Mazurie, Olaf Kniemeyer, Robert A Cramer

**Affiliations:** 1 Department of Immunology and Infectious Disease, Montana State University, Bozeman, MT, USA; 2 Leibniz-Institut für Naturstoff-Forschung und Infektionsbiologie-Hans-Knöll-Institut (HKI) Jena, Germany; 3 Institut für Mikrobiologie an der Friedrich-Schiller Universität Jena, Germany; 4 Bioinformatics Core, Montana State University, Bozeman, MT, USA

## Abstract

**Background:**

*Aspergillus fumigatus *is a mold responsible for the majority of cases of aspergillosis in humans. To survive in the human body, *A. fumigatus *must adapt to microenvironments that are often characterized by low nutrient and oxygen availability. Recent research suggests that the ability of *A. fumigatus *and other pathogenic fungi to adapt to hypoxia contributes to their virulence. However, molecular mechanisms of *A. fumigatus *hypoxia adaptation are poorly understood. Thus, to better understand how *A. fumigatus *adapts to hypoxic microenvironments found *in vivo *during human fungal pathogenesis, the dynamic changes of the fungal transcriptome and proteome in hypoxia were investigated over a period of 24 hours utilizing an oxygen-controlled fermenter system.

**Results:**

Significant increases in transcripts associated with iron and sterol metabolism, the cell wall, the GABA shunt, and transcriptional regulators were observed in response to hypoxia. A concomitant reduction in transcripts was observed with ribosome and terpenoid backbone biosynthesis, TCA cycle, amino acid metabolism and RNA degradation. Analysis of changes in transcription factor mRNA abundance shows that hypoxia induces significant positive and negative changes that may be important for regulating the hypoxia response in this pathogenic mold. Growth in hypoxia resulted in changes in the protein levels of several glycolytic enzymes, but these changes were not always reflected by the corresponding transcriptional profiling data. However, a good correlation overall (R^2 ^= 0.2, p < 0.05) existed between the transcriptomic and proteomics datasets for all time points. The lack of correlation between some transcript levels and their subsequent protein levels suggests another regulatory layer of the hypoxia response in *A. fumigatus*.

**Conclusions:**

Taken together, our data suggest a robust cellular response that is likely regulated both at the transcriptional and post-transcriptional level in response to hypoxia by the human pathogenic mold *A. fumigatus*. As with other pathogenic fungi, the induction of glycolysis and transcriptional down-regulation of the TCA cycle and oxidative phosphorylation appear to major components of the hypoxia response in this pathogenic mold. In addition, a significant induction of the transcripts involved in ergosterol biosynthesis is consistent with previous observations in the pathogenic yeasts *Candida albicans *and *Cryptococcus neoformans *indicating conservation of this response to hypoxia in pathogenic fungi. Because ergosterol biosynthesis enzymes also require iron as a co-factor, the increase in iron uptake transcripts is consistent with an increased need for iron under hypoxia. However, unlike *C. albicans *and *C. neoformans*, the GABA shunt appears to play an important role in reducing NADH levels in response to hypoxia in *A. fumigatus *and it will be intriguing to determine whether this is critical for fungal virulence. Overall, regulatory mechanisms of the *A. fumigatus *hypoxia response appear to involve both transcriptional and post-transcriptional control of transcript and protein levels and thus provide candidate genes for future analysis of their role in hypoxia adaptation and fungal virulence.

## Background

The frequency of invasive fungal infections (IFIs) has increased among immunosuppressed patient populations with the mold *Aspergillus fumigatus *the second most frequent cause of IFIs [1]. As the use of immunosuppressive therapy is increasingly common for many medical conditions, continued increases in IFI incidence are expected. While the introduction and increased use of new triazoles such as posaconazole and voriconazole have improved patient outcomes, mortality from invasive aspergillosis (IA) remains high [2-5]. Given the relatively recent emergence of these infections, molecular mechanisms of IA pathogenesis and other forms of aspergillosis are poorly understood. In theory, a better understanding of IA pathogenesis should lead to an improvement in patient outcomes through better diagnosis and use of existing therapeutics. One research area with promise for improving patient outcomes is the study of infection site microenvironment conditions on the expression of fungal virulence and *in vivo *growth factors. Recently, we observed that infection site microenvironments in the lung of IA murine models are characterized in part by hypoxia [6,7]. As oxygen is a critical component of many essential biochemical processes in eukaryotes, it has been hypothesized that the ability to overcome hypoxia is a key virulence attribute of human pathogenic fungi [8-15]. Thus, several studies in the human pathogenic yeast *Candida albicans *and *Cryptococcus neoformans *have examined the global fungal transcriptome response to hypoxia in order to better understand how human pathogenic fungi adapt to oxygen limitation [11,14,16,17]. However, the global transcriptome response to hypoxia in the pathogenic mold *A. fumigatus *has not been previously reported.

In mammalian cells, hypoxia has been observed to cause a strong and positive regulation of the transcriptome [18-20]. A key feature of the mammalian hypoxic response is the initiation of anaerobic glycolysis to maintain cellular homeostasis and regulation of glycolysis occurs both at the transcriptional and post-translational level [21]. With regard to fungi, transcriptional induction of genes in glycolysis and repression of aerobic respiration appears to be a main feature of the hypoxia response in the yeast *Candida albicans*, a facultative anaerobe [11,16,17]. However, in the obligate aerobic yeast *Cryptococcus neoformans*, a general lack of changes in glycolytic mRNA abundance was observed in response to hypoxia, and genes involved in mitochondrial function have been observed to be critical for the hypoxia response [14,22]. The effects of post-translational regulatory processes on glycolysis in *C. neoformans *are unknown. In the model obligate aerobic mold *Aspergillus nidulans*, exposure to hypoxia results in an increase in glycolytic gene transcripts, fermentation, and the GABA shunt, which bypasses two steps of the TCA cycle [23]. Transcriptome data from *A. nidulans *largely correlated with a proteomics profile where proteins in core metabolism, utilization of the GABA shunt and increases in sulfur, nucleotide and fatty acid metabolism were identified [24]. Recently, in *A. fumigatus*, glucose-limited chemostat cultures exposed to long-term hypoxia revealed 117 proteins altered in their abundance in response to hypoxia after steady-state conditions were reached [25]. Of the proteins showing a change in abundance, several were associated with glycolysis, respiration, pentose phosphate pathway, and amino acid and pyruvate metabolism. These results showed that hypoxia tends to be a positive regulator of protein expression, with 83 protein spot levels increased in response to hypoxia. Taken together, these data suggest that mechanisms of hypoxia adaptation are variable among fungi.

Here, we provide further insight into the rapid *A. fumigatus *hypoxia adaptation response by utilizing a joint transcriptomics and proteomics approach. The rationale for our study is the emerging evidence that hypoxia is a critical component of the pathogenesis of IA and other human mycoses [7,8,10,11,13,14,16,17]. Thus, understanding the molecular mechanisms of hypoxia adaptation in this human pathogenic mold will facilitate a greater understanding of aspergillosis and hopefully reveal potential areas to exploit for improving IA patient outcomes [26]. Taken together, our results reveal new insights into the molecular mechanisms of hypoxia adaptation in *A. fumigatus *that are both similar and different from previous observations in pathogenic yeast. Importantly, we present several novel candidate genes and biochemical pathways that should be examined in future experiments for their role in hypoxia adaptation and fungal pathogenesis.

## Results and Discussion

### Effect of hypoxia on transcript and protein levels in batch-fermentation culture

**Figure 1:**
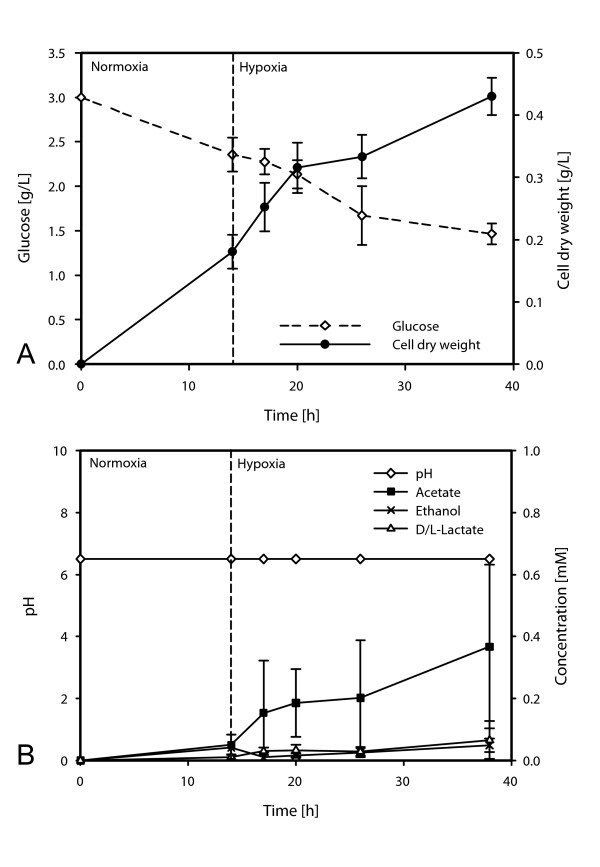
**Analysis of Growth Conditions in Oxygen-controlled Fermenter**. A) Growth (increase in mycelial dry weight) and glucose consumption (in g/L) of ***Aspergillus fumigatus ***during hypoxic cultivation in an oxygen-controlled fermenter. B) Analysis of the pH and the concentration of D/L-lactate, acetate and ethanol (in mM) in the culture supernatant. Data of three fermentation runs are shown. Three liters of AMM was inoculated with 2 × 10^9 ^conidia. After a pre-cultivation period under normoxic growth conditions (21% pO_2_) oxygen supply was shifted to low oxygen levels (0.21% pO_2_) and samples were taken after 0, 2 (microarray) or 3 (proteomics), 6, 12 and 24 hours.

To study the effect of hypoxia on the transcriptome and proteome of *Aspergillus fumigatus *two wild type strains, CBS144.89 (transcriptomics) and ATCC 46645 (proteomics) were cultured in an oxygen-controlled fermenter with glucose as the sole carbon source. After pre-cultivation under normoxic conditions (21.0% O_2_) for 14 hours the oxygen partial pressure was shifted to 0.21% O_2 _and samples were taken at 0, 2, 6, 12, and 24 hours after exposure to hypoxia. Of note, all transcript and protein level data for each time point is relative to time 0. For the first proteomics time point in hypoxia, we elected to wait one hour compared to the transcriptome time point. During cultivation, the pH of the growth medium remained constant and fungal growth occurred under both normoxic and hypoxic conditions (Figure 1A). While growth continued under hypoxia, it is clear that a reduction in growth rate occurred in response to hypoxia (Figure 1A). We elected to allow glucose to be consumed during the fermentation to more closely mimic what likely occurs during *in vivo *growth in the mammalian lung. However, glucose levels remained high even at the final time point (Figure 1A, consumption of half of the amount of glucose used in the medium), and thus are unlikely to have affected the growth rate in hypoxia. To determine the production of typical fermentation products, the concentration of lactate, ethanol and acetate in the culture supernatant was determined by enzymatic assays. The average D/L-lactate concentration in the supernatant from the culture at the 24-hour time point was determined to be 0.066 mM, and ethanol concentration was 0.049 mM. Acetate concentration did increase over time, and was approximately 0.4 mM at the end of the experiment (Figure 1B). Based on these data, we expect that the majority of transcript and protein level changes are due to the hypoxia response. However, we cannot rule out that some observed changes might be carbon-source mediated.

**Figure 2:**
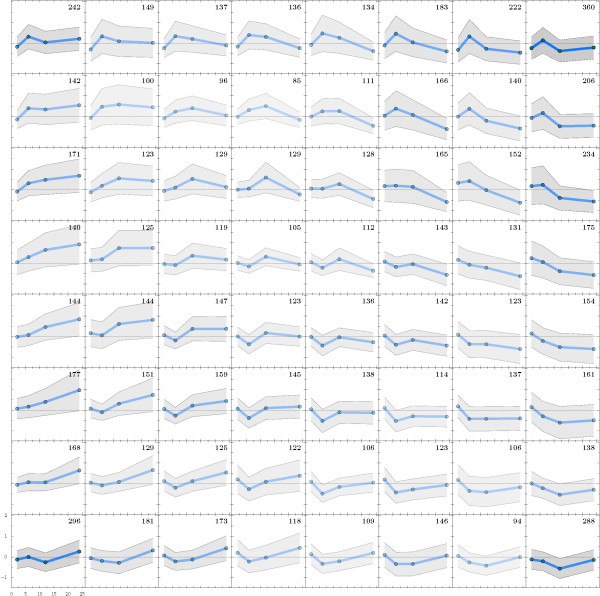
**Self Organizing Map Analysis (SOMA) of 64 clusters of transcript abundance**. This analysis shows 64 clusters of distinct patterns of transcript abundance. The highlighted square is significantly associated with ergosterol biosynthesis and TCA cycle transcripts. The transcript abundance data were clustered with a grid of size 10 × 10 (100 clusters) down to 3 × 3 (9 clusters) using the Pearson correlation coefficient as the metric between transcript profiles. The self-organizing map maximizing the number of clusters while limiting redundancies was the one of size 8 × 8 (64 clusters). Color intensity indicates the number of genes in each cluster.

Assessing the transcriptome across all time points, significance analysis of microarray (SAM) revealed that approximately 800 transcripts, or 10% of the genes on the microarray, were significantly altered in response to hypoxia (FDR < 0.05, Additional file 1). Considering all transcripts represented in the microarray, gene set enrichment analysis (GSEA) was performed to gain a general understanding of functional categories affected by hypoxia (Additional File 2). GSEA results showed that transcripts from steroid (ergosterol) biosynthesis and other metabolic processes were significantly increased during the course of the experiment. Specific categories of transcripts that were significantly increased at two hours were associated with histone deacetylases, fungal transcription factors and major facilitator superfamily genes (p < 0.01, Additional file 2). At six hours, similar transcripts were represented, but the histone deacetylases were no longer significant. Thus, it is intriguing to speculate that the early adaptation to hypoxia requires changes in chromatin structure and/or post-translational modification of key regulatory proteins. At 12 hours in hypoxia, transcriptional regulation and major facilitator superfamily transcripts continued to be increased. After 24 hours in hypoxia, few categories were significantly increased, however transcripts related to sterol related processes were highly significant. Using a self-organizing map algorithm (SOMA) to cluster genes, 64 clusters grouped the majority of steroid genes into a single cluster, highlighted in Figure 2. Thus, a major part of the *A. fumigatus *early hypoxia response is characterized by increases in ergosterol biosynthesis associated transcripts. This result is consistent with previous observations regarding the *A. fumigatus *transcriptional regulator, SrbA, which is required for growth in hypoxia and directly regulates genes in the ergosterol biosynthesis pathway [10,27].

GSEA also identified categories that are significantly reduced in response to hypoxia, and include ribosome biosynthesis, proteasome activity, pyrimidine and purine metabolism, and oxidative phosphorylation across all time points. Additionally, gene groupings associated with heat shock proteins, RNA recognition motifs, and intracellular trafficking and secretion factors were also reduced. There were fewer differences among the reduced transcripts than the increased transcripts across all time points, and more categories and domains were significant (Additional file 2). Thus, a large part of the mRNA response to hypoxia in *A. fumigatus *involves a reduction in transcript levels of genes associated with core cellular processes.

**Figure 3:**
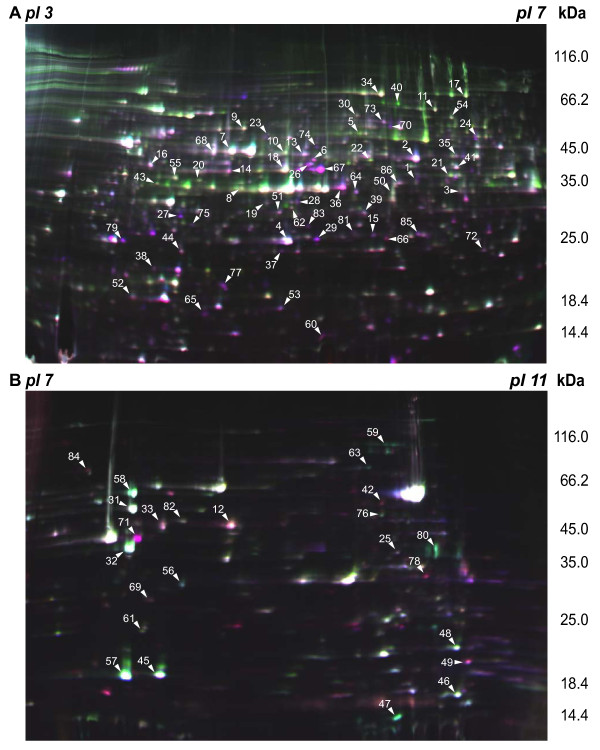
**2D gel electrophoretic separation of protein extracts of *A. fumigatus *grown under normoxic and hypoxic growth conditions**. In total, 86 different proteins of ***A. fumigatus ***changed significantly their abundance within the first 24 hours of hypoxia (protein spots are labeled with spot numbers as indicated in Additional file 3 and 5). ***A. fumigatus ***proteins were labeled with the CyDye DIGE Fluor minimal dye labeling kit. Subsequently, proteins were separated by 2D gel electrophoresis using immobilized pH gradient strips with a pH range of (A) 3-7 NL and (B) 7-11 NL in the first dimension. For the separation of proteins in the second dimension, SDS-polyacrylamide gradients gels (11-16%) were used. Differentially regulated proteins were identified by MALDI-TOF/TOF analysis. A three color overlaid gel image is shown. Samples were labeled as follows: 0 hour control sample (Cy5), 24 hour hypoxia sample (Cy3) and internal standard (Cy2).

With regard to the proteomic response of *A. fumigatus *to hypoxia, we analyzed the mycelial proteome of *A. fumigatus *wild type strain ATCC 46645 after 0 (21% pO_2_), 3, 6, 12 and 24 h growth under hypoxic conditions (0.21% pO_2_). The use of ATCC46645 allowed us to compare our results with a previous study on the proteomic changes during long-term adaptation to low oxygen levels using chemostat culture [25]. To obtain a high spatial resolution of protein spots we analyzed the intracellular proteins by two dimensional gel electrophoresis using two different immobilized pH gradient strips covering an acidic (pH 3-7) and basic pH range (pH 7-11), respectively. In total 86 differentially regulated proteins could be identified by comparing gels from different time points of hypoxic cultivation (Figure 3, Additional file 3). Under hypoxia, 52 different proteins showed an increase in abundance, whereas 34 proteins showed a decrease. Changes in protein levels occurred as early as 3 hours of hypoxic cultivation, indicating that the cellular response to hypoxia is rapid.

Looking at different primary metabolic pathways and performing protein set enrichment analysis with KEGG categories, growth under hypoxia resulted in an increased level of proteins involved in glycolysis, ethanol fermentation, electron transport, alanine, aspartate, glutamate metabolism and the oxidative stress response. In contrast, the abundance of several enzymes in the TCA cycle, the pentose phosphate shunt, and cysteine/methionine metabolism decreased during hypoxic growth conditions. Furthermore, a decreased level of proteins involved in ribosome biogenesis, sulfate assimilation and purine metabolism was also observed.

### Correlation of Proteomics and Gene Expression

**Figure 4:**
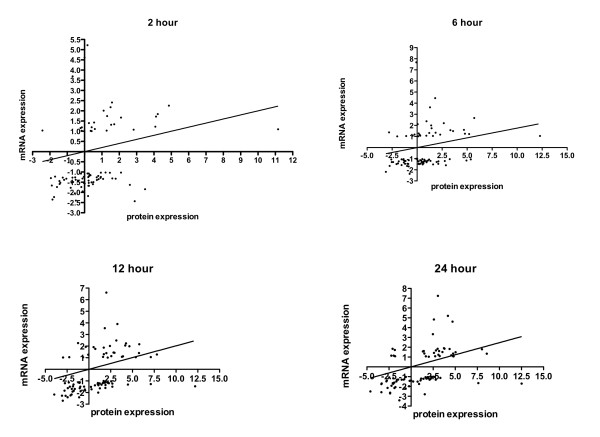
**Linear regression of protein level and mRNA level**. Slope and regression values are shown for all time points. Values are generally within correlated quadrants. Statistical analysis of the correlation between protein and microarray experiments shows a consistent trend of correlation among the datasets. Linear regressions were forced with X and Y-intercepts at zero, and varied between 0.18 and 0.25, with p < 0.05 for all correlations.

**Figure 5:**
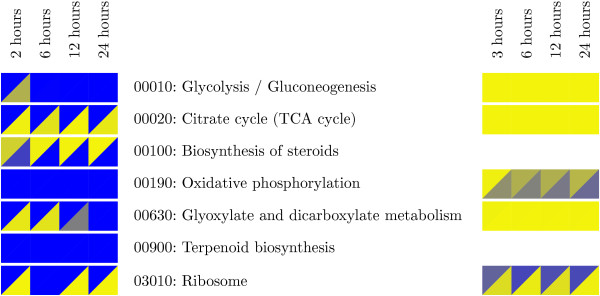
**GSEA results for both transcriptomic and proteomic data**. KEGG pathways identified as affected by hypoxia are shown. A heat map summarizes the differences and similarities observed between protein (4 columns on left) and transcript data (4 columns on right) for selected pathways. Upper-left triangles are for over-representation of increased transcript levels; lower-right triangles are for over-representation of decreased transcript levels. Yellow indicates significance for over representation, blue indicates that the transcripts are not statistically over-represented.

Proteomic analysis was performed under similar conditions as the transcriptomic analysis, however with a different wild type strain and one different early time point was analyzed. Despite the difference in wild-type strains, a correlation was observed between the proteomic and transcriptomic data. 86 protein spots showed significant changes in abundance among all experiments. Of the 86 proteins, 85 were identified in the transcriptomics data. Statistical analysis of the correlation between protein and microarray experiments shows a consistent trend of correlation among the datasets (Figure 4). Linear regressions were forced with X and Y-intercepts at zero, and varied between 0.18 and 0.25, with p < 0.05 for all correlations. A heat map summarizes the differences and similarities observed between protein and transcript data for selected pathways (Figure 5). No protein data was obtained for the terpenoid or steroid biosynthesis pathways. Additionally, glycolysis was generally not significantly over represented among either increased or decreased transcripts, however it was significant for the protein dataset. The TCA cycle and glyoxylate and dicarboxylate metabolism were decreased significantly in the transcript data, whereas both categories were significantly positively and negatively changed for the protein data. Only ribosome biogenesis was significantly down for both the transcript and protein data.

**Figure 6:**
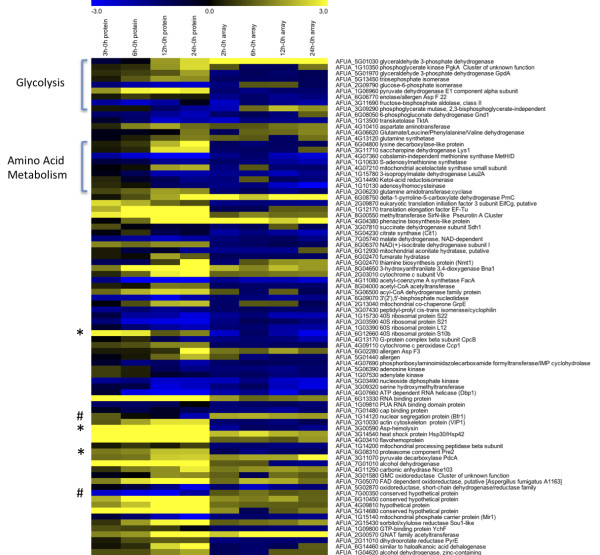
**Heat map comparison of abundance levels for both transcriptomic and proteomic data**. Differences and similarities between protein and mRNA levels are shown, blue indicates decreased levels, and yellow indicates increased levels. Data are sorted in the same order as additional file 4 in order of function. Highlighted with brackets are transcripts and proteins associated with glycolysis and amino acid metabolism, which showed different values for transcript and protein abundance. Additional differences are highlighted with # (transcript higher than protein) or * (protein higher that transcript).

Of note, in the correlation of proteomics and transcriptomics data (Figure 4), we observed very few points located in the top-left quadrant, and therefore very few instances occurred where mRNA levels increased and protein levels decreased. Two examples of this are represented by transcripts that were increased for the entire time course, yet the proteins showed decreased levels: a nuclear segregation protein Brf1 (Afu1g14120) and a hypothetical protein (Afu7g00350) (marked with # in Figure 6). In general, if the mRNA abundance was enhanced, the protein levels were also high. However, there were exceptions with genes that had reduced mRNA levels and enhanced protein abundance. Comparing the fold change values for proteins with the microarray data, 25 of the genes show different patterns of transcript and protein levels (Additional file 4). Two functional categories, glycolysis and amino acid metabolism, are noted on the heat map of comparison (Figure 6). Several of the transcripts in these groups are decreased in abundance, while the protein levels are increased. Three additional pairs are noted with an asterisk, a 40S ribosomal protein (Afu6g12660), the Asp-hemolysin (Afu3g00590) and the proteasome component Pre2 (Afu6g08310). It is possible that enhanced protein levels could be achieved through mRNA stabilization and differences in turnover rates, and it seems clear that in addition to the apparent transcript regulation of the hypoxia response, other post-transcriptional and post-translational regulatory mechanisms are active. Further identification of these regulatory mechanisms could identify new targets for gene replacement studies to determine whether these mechanisms are critical for *in vivo *growth of the fungus during hypoxia adaptation and pathogenesis.

### Comparison of the proteomic response of A. fumigatus to short- and long-term incubation under hypoxic conditions

In a previous study we analyzed the proteome of *A. fumigatus *cultivated in an oxygen-controlled, glucose-limited chemostat under normoxic (21% pO_2_) and hypoxic (0.2% pO_2_) growth conditions. In contrast to the experimental set-up chosen for the work described here, the fungus was cultivated under conditions of glucose depletion for a long time period (10 days), and the medium was exchanged continuously. Only one third of the differentially regulated proteins found in our study were also found to be differentially expressed in chemostat cultures [25]. Under both conditions, exposure of *A. fumigatus *to hypoxia caused an increase in the abundance of glycolytic enzymes and the NO-detoxifying flavohemoprotein Afu4g03410, whereas fatty acid metabolism associated protein levels were reduced. In contrast, the level of proteins associated with the pentose phosphate pathway and the citric acid cycle decreased during short-term incubation at low oxygen-levels, but increased after long-term exposure. The short-term response to hypoxia was also characterized by the activation of ethanol fermentation (see below), which could not be observed after cultivation of *A. fumigatus *in a chemostat. Furthermore, in comparison to the previous report the pseurotin A cluster was not significantly induced during the short-term exposure to hypoxia. It is interesting to speculate that either the formation of pseurotin A is only activated after longer periods of hypoxia, or glucose depletion may be a factor for derepression of the pseurotin A biosynthesis gene cluster [25].

### Glycolysis and Fermentation

**Figure 7:**
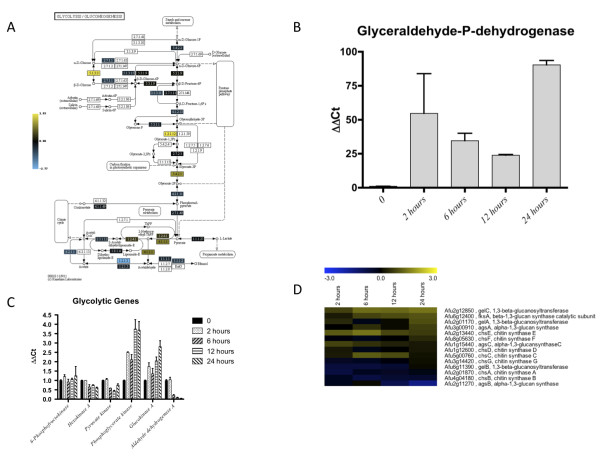
**Hypoxia affects transcript levels of enzymes involved in glycolysis consistent with fermentation and cell wall components**. A. KEGG pathway heat map representation of genes involved in glycolysis. Microarray datasets include three biological and two technical replicates. Microarrays compare wild type ***Aspergillus fumigatus ***strain CBS144.89 at the indicated times after exposure to hypoxic conditions to the time point immediately prior to hypoxia exposure (0 hours) using the median value from all replicates. Yellow indicates transcript level is higher in hypoxia. Scale indicates degree of change. B. RT-PCR of Glyceraldehyde-3-phosphate-dehydrogenase supported the observation that this was one of the most abundant hypoxia responsive transcripts in the microarray, as well as in the proteomics analysis. C. Six genes in the glycolysis pathway in ***Aspergillus fumigatus***. Transcript levels of 6-phosophofructokinase, hexokinase A and pyruvate kinase were not dramatically altered. Transcript levels of phosphoglycerate kinase and glucokinase A are 2 to 4-fold increased in response to hypoxia. Aldehyde dehydrogense A transcript level was significantly decreased at 6, 12 and 24 hours. All RT-PCRs were performed on BioRad MyIQ real-time PCR detection system with IQ SYBR green supermix. The ΔΔC**_t _**method was used to combine all biological and technical replicates for each transcript, using β-tubulin as the housekeeping gene for comparison. D. Heat map representation of cell wall component transcripts that were compiled from literature [36]. Both microarray datasets include three biological and two technical replicates, and compare wild type ***Aspergillus fumigatus ***strain CBS144.89 at the indicated times after exposure to hypoxic conditions to the time point immediately prior to hypoxia exposure (0 hours) using the median value from all replicates. Yellow indicates transcript level is higher in hypoxia.

Previous work with *A. nidulans *and *C. albicans *suggested that transcripts encoding enzymes of glycolysis are strongly induced in response to hypoxia [11,16,17,23]. With regard to *A. fumigatus*, the majority of glycolytic transcripts in the microarray were unaffected by hypoxia which is similar to observations in *Cryptococcus neoformans *(Figure 7A) [14]. However, increases in glycolytic protein levels were observed from the proteomics data. Taken together, these data suggest that the primary regulation of glycolysis in response to hypoxia in *A. fumigatus *may occur post-transcriptionally. Intriguingly, the glycolytic transcript most induced by hypoxia in *A. fumigatus *was the glyceraldehyde-3-phosphate dehydrogenase (GAPDH) Afu5g01030, and this was verified with RT-PCR (Figure 7B). This enzyme is one of three GAPDHs in the genome of *A. fumigatus *that likely catalyzes the 6^th ^step in glycolysis. The other two GAPDHs, Afu5g01970 and Afu8g02560, were slightly negatively altered in transcript abundance (Additional file 5). The increase in mRNA expression of Afu5g01030 was further validated by proteomics, where this was one of the most abundant proteins in hypoxia (Additional file 3). It is likely important that this glycolytic gene appears to be regulated at the level of transcription unlike other glycolytic genes in *A. fumigatus *[28]. In the genome of *S. cerevisiae*, three GAPDH genes also are found, and these genes show different growth-phase dependent regulation. Most interestingly, the corresponding proteins are not only located in the cytosol but also present in the cell wall. Thus, it has been speculated that they mediate cell adhesion [29]. Whether additional roles for GAPDHs in fungal biology and pathogenesis exist remain to be determined.

Also related to glycolysis, both transcripts that are required for the conversion of pyruvate to ethanol are induced in response to hypoxia: *pdcA *(Afu3g11070) encoding a pyruvate decarboxylase, is one of the most highly induced transcripts, along with two alcohol dehydrogenases, *alcC *(Afu5g06240) and *adh2 *(Afu2g10960). PdcA and AlcC were both recently confirmed to be required for ethanol fermentation in *A. fumigatus*, and AlcC was demonstrated to have an important but undefined role in fungal pathogenesis [7]. Consistent with the transcript level results, the protein level of pyruvate decarboxylase PdcA was increased in hypoxia (Additional File 5). In addition, the observed increase in ethanol fermentation is in contrast to the previous proteomic analysis of the *A. fumigatus *hypoxic response, which did not find evidence of an NAD^+ ^regenerating system [25]. This is likely explained by the different growth conditions used between the two studies. However, our results here with the short term hypoxia response are consistent with recent findings in a murine model of IPA that suggest ethanol fermentation is part of *in vivo *growth mechanisms of *A. fumigatus *[7]. In addition, in *A. nidulans*, an ethanol fermentation response to hypoxia was also observed [23,24]. However, loss of ethanol fermentation genes in *A. fumigatus *did not dramatically affect the *in vitro *growth rate of the fungus in hypoxic conditions. Thus, other mechanisms of NAD^+ ^regeneration are likely present in *A. fumigatus *and it is unclear what role ethanol fermentation plays in the hypoxia response. One possible alternative fermentation mechanism is lactate fermentation as has been observed in *A. nidulans*. In support of this hypothesis, a mitochondrial lactate dehydrogenase transcript (Afu1g00510) was transcriptionally increased in response to hypoxia. However, the determined lactate concentration in the culture supernatant was only in the micromolar range (see Figure 1B). Thus, the role of fermentation and NAD^+ ^regeneration in the *A. fumigatus *hypoxia response awaits further mechanistic characterization.

In contrast to transcript levels, high protein levels of AlcA (Afu7g01010) suggest a critical role for this protein in the hypoxia response, as it was one of the highest induced proteins (Additional files 3 and 4). In *A. nidulans*, AlcA is involved in utilization of ethanol as a carbon source [30] and in accordance with this, the level of the AlcA protein highly increased during growth of *A. fumigatus *on ethanol as shown by a 2D gel electrophoresis study [31]. Thus, it is possible that as ethanol production increases in response to hypoxia, *A. fumigatus *is then able to utilize the produced ethanol as a carbon source, which may have potential implications for fungal pathogenesis. Alternatively, as these experiments were conducted in a glucose rich environment, the role of ethanol fermentation and/or utilization in the hypoxia response may be overstated and depend on the availability of fermentable substrates.

To further support the observed changes in transcript levels from the microarray data, we chose six transcripts involved in glycolysis for additional analyses using real-time RT-PCR (Figure 7C). Glycolysis starts with the conversion of D-glucose to D-glucose 6-phosphate by hexokinase or glucokinase. There are three hexokinases annotated in the KEGG database (Afu2g00450, Afu2g05910, and Afu6g03980) in *A. fumigatus*, and these tended to be transcriptionally reduced or unchanged in response to hypoxia, whereas the two glucokinases (Afu6g02230 and Afu2g16330) were induced at later time points when glucose levels began to drop. Thus, it is unclear whether these glucose responsive transcripts were altered due to hypoxia or changes in the glucose level in the culture medium at later time-points. We chose to look at Afu6g02230 (*glkA*) and Afu2g05910 (*hxkA*) as these are thought to be the main active enzymes in *A. fumigatus *for this part of glycolysis [32]. As predicted by the microarray data, the *hxkA *transcript was repressed and the *glkA *transcript was enhanced. This regulation may be explained either by a stress-induced expression of *hxkA *[33] or by an increase in transcript levels of the high-affinity sugar kinase *glkA *when the glucose concentration decreases.

Afu4g00960 transcript, 6-phosphofructokinase, one of the early steps in converting glucose 6-phosphate to glyceraldehyde 3-phosphate was slightly reduced in the microarray, and relatively unchanged in PCR. Afu1g10350, phosphoglycerate kinase (*pgkA*) at the midpoint of the glycolytic pathway was not significantly changed in the microarray. However, RT-PCR data suggest that this transcript is increased in response to hypoxia, which is consistent with findings in mammals for this transcript [34]. Proteomic data confirms the PCR values and shows that PgkA protein levels were also enhanced in response to hypoxia (Additional files 3 and 4). Afu6g07430 transcript, a putative pyruvate kinase, is the final step in the glycolytic pathway and transcript levels were reduced in the microarray and verified with RT-PCR (Figure 7C). However, changes in protein levels of this enzyme were not detected.

Another gene associated with glycolysis investigated with qRT-PCR was Afu6g11430 (*aldA*), aldehyde dehydrogenase A, which is necessary for conversion of acetaldehyde to acetate for central carbohydrate and lipid metabolism. Microarray results suggest that this transcript was reduced in response to hypoxia and RT-PCR results confirm this observation (Figure 7C). However, there are four additional aldehyde dehydrogenase encoding genes in *A. fumigatus*, Afu2g00720 and Afu7g01000 (NAD^+^) and Afu4g08600 and Afu4g13500 (NAD(P)^+^), so it is possible that these enzymes, and not AldA, are utilized under hypoxic conditions to metabolize ethanol. Afu4g08600 does show a gradual increase in transcript by 24 hours in hypoxia when ethanol levels are assumed to be higher, whereas Afu7g01000 and Afu4g13500 transcripts are reduced, although not to the same degree as *aldA *(Additional file 5). A recent proteomics study indicated that Afu7g01000 is most probably the major aldehyde dehydrogenase involved in the metabolism of ethanol, as it was highly expressed in *A. fumigatus *during growth on ethanol [31]. Afu2g00720 was not on the microarray. Taken together, these results suggest that hypoxia plays a small role in altering the transcription of glycolysis encoding enzymes, however, the increase in protein levels of several enzymes coupled with the apparent increase in fermentation suggest that glycolytic activity is likely increased in response to hypoxia in *A. fumigatus *and regulation may occur at posttranscriptional levels. This observation confirms a previous study, which showed post-transcriptional regulation of glycolysis in anaerobic *S. cerevisiae *cultures [35].

Of critical relevance to fungal pathogenesis, cell wall biosynthesis transcripts are affected by hypoxia (Figure 7D) [36]. Specifically, the 1,3-β-glucan synthase *fksA *(Afu6g12400) and the 1,3-α-glucan synthase *agsA *(Afu3g00910) are increased along with transcripts for glucanosyltransferases *gelC *(Afu2g12850) and *gelA *(Afu2g01170). It is also noted that chitin synthases are altered in expression. Specifically, *chsE *(Afu2g13440) and *chsF *(Afu8g056300) transcripts are increased, while the remainder of chitin synthase transcripts are unchanged or reduced. Many cell wall polymers require precursors from the glycolysis pathway and thus alterations in glycolytic flux in response to hypoxia may alter available cell wall precursor levels. Taken together, these results suggest that cell wall composition changes occur in response to hypoxia, which has important ramifications for the effect of cell wall-targeting antifungals, host immune system recognition of *A. fumigatus*, and the development of the inflammatory response to the invading fungus.

### Ergosterol Biosynthesis

**Figure 8:**
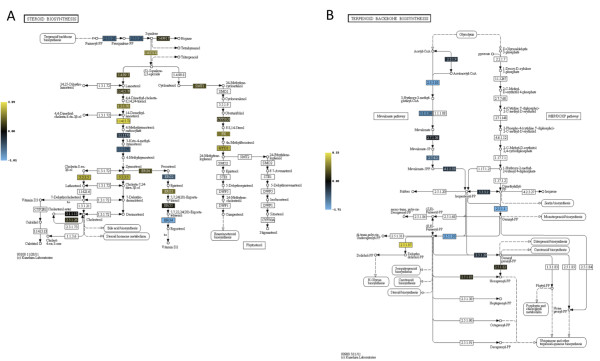
**Hypoxia increases transcript levels of enzymes involved in terpenoid and ergosterol biosynthesis**. A. KEGG heat map representation of genes involved in steroid (ergosterol) biosynthesis and B. terpenoid (isoprenoid) biosynthesis. The median value from three biological replicates was used. If multiple transcripts exist for a given enzymatic step (***i.e. ***HmgA and HmgB for 2.3.3.10), the transcript with the highest amplitude was used. Microarray data compares mRNA levels of wild type ***A. fumigatus ***at the indicated times after exposure to hypoxic conditions to time point immediately prior to hypoxia exposure (0 hours). Yellow indicates transcript levels are increased compared to normoxia (0 hours). Blue indicates transcript levels are reduced compared to normoxia (0 hours).

Ergosterol is the fungal equivalent of mammalian cholesterol and in addition to being a critical component of plasma membranes, is also a target of the most commonly used antifungal drug against *A. fumigatus*, the triazole voriconazole. Importantly, previous research has identified sterols as an oxygen sensing system in fungi including *Saccharomyces cerevisiae *and *Schizosaccharomyces pombe *[37,38]. This is likely due to the large oxygen requirement for sterol biosynthesis. Previous transcript profiling experiments of hypoxia responses in the human fungal pathogens *C. neoformans *and *C. albicans *identified the ergosterol biosynthesis pathway as one of the most affected by hypoxia [14,16]. In addition, we previously observed a significant reduction in several ergosterol biosynthesis enzyme-encoding transcripts in response to hypoxia in the *A. fumigatus *SREBP null mutant (SrbA, Afu2g01260) [10]. Thus, it is not surprising that one of the most affected metabolic pathways in our transcript profiling experiments in this study was steroid biosynthesis. To fully explore the observed significance of steroid biosynthesis genes, we compiled a complete list of ergosterol genes from previously published work that includes the entire pathway from acetyl-CoA to ergosterol, including terpenoid backbone biosynthesis [39]. Transcripts of ergosterol biosynthesis enzymes previously shown to require oxygen were highly induced in response to hypoxia and largely clustered together, particularly the C-14 sterol reductases *erg24A *(Afu1g03150) and *erg24B *(Afu1g05720), and the C-4 methyl sterol oxidases *erg25A *(Afu8g02440) and *erg25B *(Afu4g04820), which are all enzymes needing three molecules of oxygen for full function (Figure 8A) [40]. Unfortunately, another oxygen dependent enzyme, the target of the triazole drugs, Erg11A (Cyp51A, Afu4g06890) was not on the microarray. However, previous work has established that *erg11A *transcript is generally increased in response to hypoxia [41].

In addition to these, several other ergosterol biosynthesis genes are duplicated in the *A. fumigatus *genome. The three copies of sterol desaturases, *erg3A *(Afu6g05140), *erg3B *(Afu2g00320) and *erg3C *(Afu8g01070), convert episterol to 5,7,24(28)-ergostatrienol, and require oxygen and NADPH [42]. *Erg3A *and *erg3B *are significantly increased at all time points, whereas the *erg3C *transcript is slightly decreased. The two copies of C-24(28) sterol reductase genes, *erg4A *(Afu5g14350) and *erg4B *(Afu1g07140), are the last step of ergosterol biosynthesis, and both show decreased transcript levels in response to hypoxia. Three copies of the lanosterol cyclase, *erg7A *(Afu5g04080), *erg7B *(Afu4g012040) and *erg7C *(Afu4g14770), are involved in the transition from epoxysqualene to lanosterol. All transcripts are increased, and most significantly at 6 and 12 hours. *Erg7A *and *erg7B *are slightly decreased in expression at 24 hours. The two copies of acetyl-CoA-acetyltransferase, *erg10A *(Afu6g14200) and *erg10B *(Afu8g04000) are relatively unchanged in expression over the 24-hour period of hypoxia exposure. These enzymes are at the earliest stage of the terpenoid biosynthesis pathway, converting 2 acetyl-CoA to acetoacetyl-CoA. This important pathway is upstream of the ergosterol pathway and condensation of terpenoid building blocks (five-carbon isoprene units) leads to precursors for sterol biosynthesis. Next, *erg13A *(Afu8g07210) and *erg13B *(Afu3g10660) are significantly decreased, with *erg13B *more so than *erg13A*. This is the next step in the terpenoid biosynthesis pathway, converting acetoacetyl-CoA to 3-hydroxy-3-methyl-glutaryl-CoA. Finally, the next step in the terpenoid pathway converts 3-hydroxy-3-methyl-glutaryl-CoA to mevalonate. HMG-CoA reductase (HMGR) is the rate-limiting step in ergosterol biosynthesis and both *hmgA *(Afu2g03700) and *hmgB *(Afu1g11230) have reductions in transcript levels in response to hypoxia. Generally, we observe that the duplicated genes in the ergosterol pathway share similar, although not identical, patterns of expression in response to hypoxia.

An intriguing question is the mechanism behind the increase of specific ergosterol biosynthesis transcripts in response to hypoxia. One mechanism is thought to be through direct transcriptional regulation by the fungal SREBP transcription factor, which has been shown to be a key transcriptional regulator of ergosterol biosynthesis [10,13,14,27,43]. For *S. pombe*, activation of the SREBP Sre1 by proteolytic cleavage has been linked to sensing of total ergosterol levels in the cell [44]. As sterol biosynthesis decreases upon reduction of available oxygen, the SREBP pathway senses the drop in total ergosterol levels and is capable of restoring sterol homeostasis through induction of sterol biosynthesis genes [44]. However, another mechanism may be suggested in our transcript profiling data, which illustrates a reduction in transcripts associated with terpenoid biosynthesis, which lies upstream of ergosterol production.

As previously stated, several transcripts in the terpenoid (isoprenoid) backbone biosynthesis pathway are reduced in response to hypoxia (Figure 8B). All three initial steps in the terpenoid/mevalonate biosynthesis pathway (*erg10, erg13 *and *hmg*) have reductions in transcript levels in response to hypoxia (Figure 8B). Thus, a decrease in needed precursors, such as mevalonate, for sterol biosynthesis may also stimulate an increase in transcript levels of enzymes further down the pathway in response to the growing reduction in important precursor levels. The terpenoid (isoprenoid) biosynthesis pathway also leads to N-glycan and ubiquinone and other terpenoid-quinone biosynthesis pathways in *A. fumigatus*. Moreover, mevalonate was shown to be a key intermediate in the biosynthesis of siderophores in *A. fumigatus *under iron-limited conditions [45]. Transcripts in the N-glycan biosynthesis pathway remain mostly reduced in hypoxia, with the exception of three genes: Afu2g14630, cell wall glycosyl hydrolase; Afu6g04210, mannosyl-oligosaccharide glucosidase; and Afu6g09770, geranylgeranyl diphosphate synthase (Additional files 2 and 5). Transcripts associated with ubiquinone biosynthesis are also slightly reduced, but mostly remain unchanged (Additional file 2). Thus, only transcripts in the ergosterol biosynthesis pathway are significantly increased downstream of the terpenoid biosynthesis pathway. A direct regulatory link between terpenoid and ergosterol biosynthesis remains unclear. Most likely, HmgR plays a significant role in coordinately altering the biosynthetic activity of these pathways [46].

### Iron acquisition and siderophore biosynthesis

**Figure 9:**
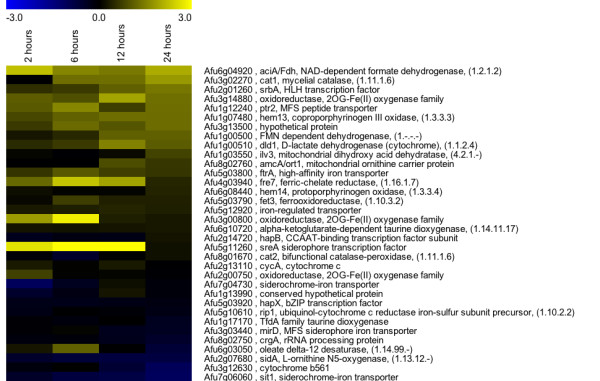
**Hypoxia increases transcript levels of enzymes involved in heme biosynthesis, iron-associated and SreA-associated processes**. Microarray datasets include three biological and two technical replicates to create heat maps using a median values to compare wild type *Aspergillus fumigatus *strain CBS144.89 at the indicated times after exposure to hypoxia to time point immediately prior to hypoxia exposure (0 hours). Yellow indicates transcript level is higher under exposure to hypoxia. Data are sorted by the late time point (24 hours post exposure to hypoxia).

Taking only transcripts that were most significantly changed in abundance using SAM (Additional file 1), analysis with FungiFun https://sbi.hki-jena.de/FungiFun/FungiFun.cgi showed additional significant categories. In addition to the previous pathways identified by GSEA, oxidation-reduction and iron related transcripts are significantly increased in response to hypoxia (Additional file 6). Importantly, iron is a required cofactor for many of the transcripts coding for enzymes associated with ergosterol biosynthesis steps that were increased in response to hypoxia (*erg*25 and *erg*11). A literature search provides a list of iron-related transcripts to investigate the microarray results [47-49]. The majority of transcripts associated with iron acquisition and biosynthesis are somewhat increased in response to hypoxia (Figure 9). However, transcripts related to siderophore biosynthesis (i.e. *sidA*, Afu2g07680) and iron uptake (i.e. *sit1*, Afu7g06060) were slightly transcriptionally reduced. SreA (Afu5g11260), a previously identified negative regulator of iron homeostasis, showed increased transcript levels at 2, 6, and 12 hours, so that the iron-limitation response was apparently not induced under the tested conditions. Importantly, we must distinguish *A. fumigatus *SreA from SrbA the sterol regulatory element binding protein in this mold (SREBPs in yeast have been named Sre1). There is also an apparent decrease in terpenoid intermediates in response to hypoxia which may negatively affect siderophore biosynthesis [45]. A further discussion on the implications in changes in iron homeostasis mechanisms is below in the transcription factor analysis. In general, these results are consistent with a requirement for iron in the hypoxia response that has been observed in mammalian systems and was shown for *A. fumigatus *when it was cultivated in a glucose-limited chemostat at low oxygen levels [21,25]. Moreover, in *C. albicans *an increase in iron uptake transcripts was also observed upon exposure to hypoxia [11,17]. Recently, we observed that SrbA also regulates iron uptake and homeostasis in iron replete conditions under hypoxia and in low iron conditions, further suggesting an important link between iron uptake and the *A. fumigatus *hypoxia response that remains to be further explored [27].

### Tricarboxylic acid cycle, GABA Shunt, and Respiration

**Figure 10:**
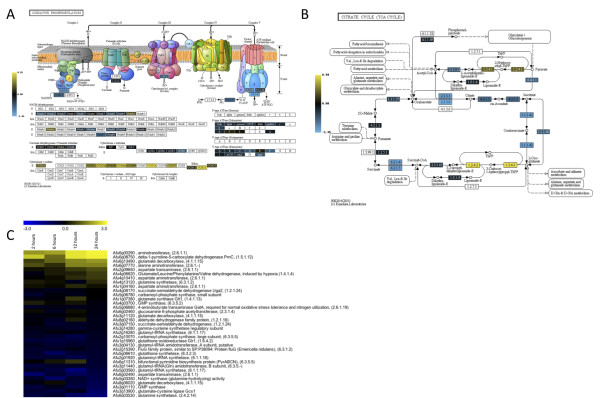
**Hypoxia affects transcript levels of enzymes involved in the tricarboxylic acid (TCA) cycle, oxidative phosphorylation, glutamate biosynthesis and the GABA shunt**. A. KEGG heat map representation of genes involved oxidative phosphorylation comparing transcript levels at the indicated times after exposure to hypoxic conditions to time point immediately prior to hypoxia exposure (0 hours). Yellow indicates transcript level is higher. Scale on left indicates intensity of expression. B. KEGG heat map representation of genes involved in the TCA cycle, comparing transcript levels at the indicated times after exposure to hypoxic conditions to time point immediately prior to hypoxia exposure (0 hours). Yellow indicates transcript level is higher. Scale on left indicates intensity of expression. C. Heat map representation of transcripts involved in glutamate biosynthesis, including transcripts associated with the GABA shunt. Both heat maps compare wild type ***Aspergillus fumigatus ***strain CBS144.89 at the indicated times after exposure to hypoxic conditions to time point immediately prior to hypoxia exposure (0 hours). Yellow indicates transcript level is higher, and values are sorted by the 24-hour time point.

For many organisms, exposure to hypoxia results in decreased flux through the TCA cycle concomitant with a decrease in aerobic respiration [16,43]. Correspondingly, most transcripts associated with oxidative phosphorylation complexes were decreased; although cytochrome C oxidase associated transcripts were overall increased (Figure 10A). Additionally, many transcripts associated with the TCA cycle were reduced in response to hypoxia at the time points we examined in our experiments (Figure 10B). In contrast, several transcripts in the TCA cycle are transcriptionally increased in response hypoxia in *A. nidulans *[23]. In addition, the industrial mold *Aspergillus niger *has been observed to increase production of TCA cycle intermediates during hypoxic cultivation [50]. However, *A. fumigatus *TCA cycle gene transcripts were largely reduced in response to hypoxia, although the conditions of the experiments were not identical to the previous *Aspergillus *experiments (Figure 10B, Additional file 5). For example, transcripts for fumarate reductase OsmA (Afu8g05530), phosphoenolpyruvate carboxykinase AcuF (Afu6g07720), and putative malate dehydrogenase homologs (Afu2g13800 and Afu7g05740) were reduced in response to hypoxia. These transcripts are key factors in the reductive branch of the TCA cycle, which in some organisms such as *Mycobacterium tuberculosis *are important for the reoxidation of intracellular NADH during hypoxic growth [51]. In further support of these data, putative succinate dehydrogenase transcripts *sdh1 *(Afu3G07810) and *sdh2 *(Afu5g10370) were also transcriptionally reduced in response to hypoxia (Additional File 4). Thus, hypoxic responses even amongst the genus *Aspergillus *are divergent, and it appears that *A. fumigatus *does not use the reductive branch of the TCA cycle to adapt to the hypoxia culture conditions utilized in our study.

As observed with *A. nidulans*, there is some evidence of the GABA shunt being utilized by *A. fumigatus *in response to hypoxia (Figure 10B, C). The GABA shunt is hypothesized to help organisms avoid the accumulation of high NADH levels in the absence of a terminal electron acceptor such as oxygen and also contributes to glutamate formation. Of the six *A. nidulans *genes identified involved in the GABA shunt [23], there are five potential homologues in *A. fumigatus*: Afu4g06620 and Afu2g06000 (glutamate dehydrogenase), Afu6g13490 (glutamate decarboxylase), Afu5g6680 (aminobutyrate transaminase, *gatA*) and Afu3g07150 (succinate-semialdehyde dehydrogenase). Of these five, four were on the *A. fumigatus *microarray, missing Afu2g06000. Transcripts for two of these genes were increased in response to hypoxia: glutamate decarboxylase Afu6g13490 and glutamate dehydrogenase Afu4g06620 (Figure 10B, C). In the proteomic data, Afu4g06620 protein levels are increased after 24 hours in hypoxia. However, this pathway has not been specifically studied in *A. fumigatus*, so it is possible that more distant homologues may function in this pathway. Moreover, with the reduction in TCA cycle transcripts, it is unclear what the available levels of 2-oxoglutarate would be to allow flux through the GABA shunt in *A. fumigatus*. Investigating the KEGG defined glutamate pathway shows that glutamate decarboxylase (Afu6g13490) is also associated with glutamate biosynthesis, and a great number of transcripts do show increased levels in this pathway in response to hypoxia (Figure 10C). Glutamate decarboxylases have a wide range of functions depending on the organism. For example, in *S. cerevisiae*, GAD1 is critical for normal tolerance to oxidative stress [52]. Thus, the increased levels of transcripts associated with the GABA shunt and glutamate biosynthesis in *A. fumigatus *may be a response to the changing redox status of the cell in response to hypoxia.

### Novel genes showing positive regulation

**Figure 11:**
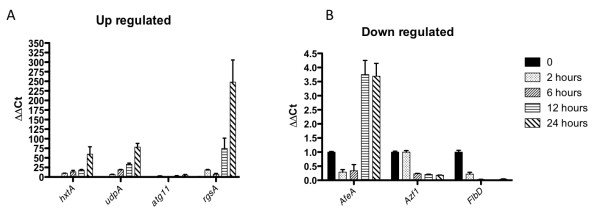
**RT-PCR of novel transcripts affected by hypoxia**. A. Four transcripts (Afu3g14170 (***hxtA***), Afu2g09590 (***udpA***), Afu3g11590 (***atg11***), Afu5g00900 (***rgsA***)) that were significantly increased using real time RT-PCR. B. Three transcripts (Afu5g12510 (***afeA***), Afu6g05160 (***azf1***), Afu1g03210 (***flbD***)), that were significantly decreased using real time RT-PCR. All reactions were performed on BioRad MyIQ real-time PCR detection system with IQ SYBR green supermix. The ΔΔC**_t _**method was used to combine three biological and 2 technical replicates for each transcript, using β-tubulin as the housekeeping gene for comparison.

Novel and interesting transcripts were increased significantly in response to hypoxia in the microarray analysis, and not associated with a KEGG pathway. Some of these transcripts were verified with RT-PCR. For example, Afu3g14170 (*hxtA*), a major facilitator superfamily glucose transporter and putative high-affinity hexose transporter, was up at all time points (Figure 11A). The homolog in *A. nidulans *is associated with nutrient starvation and sexual reproduction [53]. The homolog in *S. cerevisiae *senses low glucose, mannose and fructose, and induces hexose transporters [54]. The trend of increasing expression continues over time, consistent with reduction in glucose in the growth media and the increased mRNA levels of the glucokinase *glkA*, which is characterized by a higher affinity to glucose in comparison to the hexokinase HxtA.

Afu2g09590 (*udpA*), an UDP-N-acetylglucosamine 1-carboxyvinyltransferase family member, was strongly increased in hypoxia, and is a protein generally associated with glycan biosynthesis in bacteria, where this gene is commonly named MurA. This gene is associated with the first step in peptidoglycan biosynthesis in bacteria, and is a target for the antibacterial drug fosfomycin [55]. However, a database query http://aspgd.broadinstitute.org/cgi-bin/asp2_v3/shared/show_protein_cluster.cgi?site=asp2_v3&id=960184 shows a similar gene in several *Aspergillus *species syntenic within the inspected region. The function of this protein in fungal species is unknown.

Afu3g11590 (*atg11*) transcript was increased at all time points. The gene is homologous to autophagy related protein 11 (ATG11) in *S. cerevisiae*, and has been shown to be involved in trafficking of autophagosomes and cytoplasm to vacuole (Cvt) vesicles [56]. ATG genes are conserved among eukaryotes, however the function of the Atg11 protein appears to be fungal specific and depends on the interaction with other proteins in the autophagy pathway [57]. Autophagy is stress induced, and it is thought that it can function to recycle cellular components that are no longer necessary into pathways that are critical for survival. In this way, autophagy helps to reduce the energy costs for significant changes in cell physiology [58]. Thus, it is tempting to speculate that autophagy is one mechanism by which *A. fumigatus *adapts to hypoxic stress.

Afu5g00900 (*rgsA*), a G-protein signal regulator with an RGS domain, is predicted to be involved in attenuation of G-protein signaling through activation of the intrinsic GTPases of G-proteins [59]. This gene was one of the most highly induced genes in the microarray analysis. Although this gene has not been investigated in *A. fumigatus*, it is likely that this gene allows for the rapid response of the cell to environmental changes. In *A. nidulans*, the homologue of this gene (*rgsA*) negatively regulates the G protein α subunit GanB, which is involved in the activation of various stress responses and the inhibition of asexual conidiation. During hypoxia RgsA is putatively required to down-regulate these energy-consuming stress responses [60]. The GanB ortholog GpaB in *A. fumigatus *was shown to regulate the virulence gene *pksP *and loss of GpaB function caused an increased susceptibility to killing by macrophages [61]. In mammalian solid tumors, Rgs2 is associated with hypoxia and pro-tumor functions [18]. Rgs2 appears to be a critical regulator of the pro-angiogenic function of myeloid derived suppressor cells, and deletion of Rgs2 in a murine model significantly reduced tumor growth. Thus, Rgs2 is an intriguing candidate for future study of its role in fungal hypoxia adaptation and pathogenesis.

### Novel genes whose transcripts decrease in response to hypoxia

Analysing only genes in the SAM dataset (Additional file 1) with FungiFun, functional categories with transcripts largely reduced in abundance are similar to the GSEA analysis and include ribosome and RNA processing and the TCA cycle (Additional file 7). An additional selection of transcripts whose levels were significantly reduced in hypoxia was also validated with RT-PCR. First, Afu5g12510 (*afeA*), an adenylate-forming enzyme, was down in the 2 and 6 hour time points and up at 12 and 24 hours in the microarray experiment, and this was confirmed by PCR (Figure 11B). Adenylating enzymes generally activate otherwise unreactive carboxylic acids and can be used to form a wide array of natural products [62]. This gene is a member of the acyl-CoA synthetases (AMP-forming)/AMP-acid ligases family, and potentially involved in lipid metabolism (COG ontology). It is also identified in PANTHER as an ATP-dependent AMP-binding enzyme family member, with a 4-coumerate CoA ligase sub-family designation. Among the most highly reduced proteins, the level of the acetate activating acetyl-coenzyme-A synthetase FacA decreased most significantly in response to hypoxia. This finding suggests an oxygen-dependent regulation of the acetate-activating enzyme FacA that has also been reported for the yeast orthologue Acs1p [63]. Taken together, these results suggest that fatty acid metabolism may be reduced in *A. fumigatus *in response to hypoxia, contrary to results in other organisms such as the pathogenic yeast *C. neoformans *[14].

Next, Afu6g05160 (*azf1*) is a zinc-finger C2H2-type transcription factor, which may be involved in the regulation of cell cycle, the G2/M transition, and is activated in non-fermenting conditions in *S. cerevisiae *[64]. Consistent with this, the transcript level was reduced in microarrays after 2 hours in hypoxia, and PCR results confirmed this observation (Figure 11B). Additionally, this transcription factor is a positive regulator of the G1 cyclin CLN3 in *S. cerevisiae *in response to glucose [65]. CLN3 is involved in the progression of the cell cycle, and activates Cdc28p kinase which promotes the G1 to S phase transition [66]. In general, the transcriptomics and proteomics data along with the actual growth curve of the fungus in hypoxia all suggest a reduction in cellular metabolism, which likely would correspond to changes in the regulation of cell cycle length.

Finally, transcript levels of Afu1g03210 (*flbD*), homologous to a Myb family conidiophore development gene in *A. nidulans *[67-69], were reduced at all time points, and this was verified by PCR (Figure 11B). Therefore, it is likely that in response to hypoxia, asexual reproduction is inhibited. Consistent with this hypothesis, the target of FlbD, BrlA (Afu1g16590), is also repressed in expression in the microarrays at all time points. A bZIP protein, FlbB (Afu2g14680), which acts upstream of FlbD, is also repressed in expression, almost 2-fold by 24-hour time point (Additional file 5) [70]. Interestingly, the FlbB locus is also associated with gliotoxin production. Moreover, it is well known that *in vivo *during invasive aspergillosis that asexual reproduction generally does not occur with *A. fumigatus*. Thus, the *in vivo *hypoxic microenvironment might repress the transcriptional program needed for asexual development in response to the host environment.

### Transcriptome analysis of transcription factors

**Figure 12:**
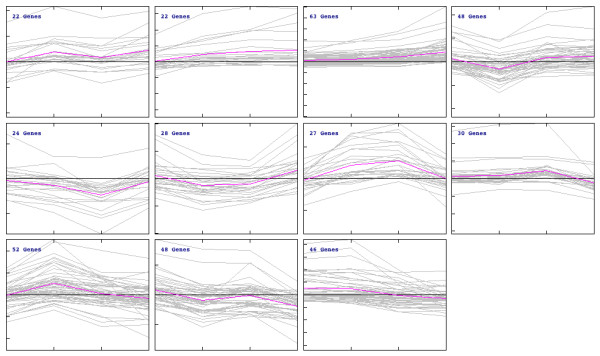
**Self organizing tree algorithm (SOTA) clusters of transcription factor transcript levels in response to hypoxia**. Eleven clusters were identified in MeV using the SOTA function. Grey lines are the individual transcription factor transcript level, and pink is the average trend line for a given cluster. The majority of transcripts are not associated with a pathway, and therefore no significant categories, other than DNA binding, were detected among the clusters. Each tick mark on the X-axis represents each time point in the experiment, and each tick mark on the Y-axis represents a fold change in transcript level. The box marked with an asterisk represents the cluster containing the known hypoxia responsive transcription factor, SrbA. These transcripts are further evaluated in Figure 13.

**Figure 13:**
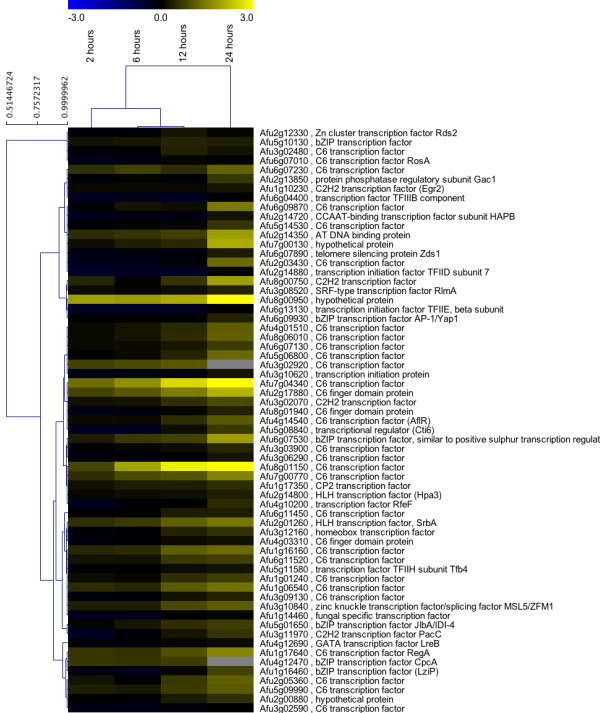
**Self-organizing tree algorithm (SOTA) cluster associated with the transcription factor SrbA**. A hierarchical tree generated from the median transcript levels of 63 transcription factors showing a similar transcript level profile with a known hypoxia responsive transcription factor SrbA identified from the SOTA analysis in Figure 12. Microarray datasets include three biological and two technical replicates. Microarrays compare wild type *Aspergillus fumigatus *strain CBS144.89 at the indicated times after exposure to hypoxic conditions to the time point immediately prior to hypoxia exposure (0 hours). Yellow indicates transcript level is higher in hypoxia.

Several significant functional categories of positively affected transcripts were associated with transcription factors (Additional file 2). To investigate this more completely, a list of 430 genes annotated as transcription factors was compiled from the genome of *A. fumigatus*. Of those, 414 were present on the array (TF tab, Additional file 5). The majority of these genes have not been directly investigated in *A. fumigatus*. Self-organizing tree algorithm (SOTA) analysis [71,72] reveals distinct patterns of changes in transcript abundance over the course of the experiment (Figure 12). Compared to other classes of genes in our analyses, transcription factors are largely positively regulated in response to hypoxia. As expected, transcript levels of a transcriptional regulator of hypoxia adaptation in *A. fumigatus*, SrbA (Afu2g01260), increased over the course of the experiment. This correlates with the observed increase in ergosterol biosynthesis gene transcripts that are likely directly regulated by SrbA [10,27,41]. The cluster associated with the SrbA transcript contains 63 transcription factors that mirror the expression pattern of a gene known to be critical for adaptation to hypoxia and virulence, thus these transcription factors may be exciting targets for gene replacement studies to determine their role in hypoxia adaptation and virulence (Figure 13).

Transcription factors involved in regulation of iron homeostasis were also responsive to hypoxic cultivation. The GATA type transcription factor SreA (Afu5g11260) has increased transcript levels at 2, 6, and 12 hours hypoxia, while HapX (Afu5g03920) transcripts are correspondingly slightly reduced. This is consistent with SreA's role as a repressor of HapX [73,74]. The transcript profile of these two key iron regulators suggests that HapX may be slowly released from SreA repression as the exposure to hypoxia increases. This would be consistent with hypoxic cells initially being iron replete, but as the iron supplies become utilized by key pathways required for hypoxia adaptation (ergosterol biosynthesis, respiration), SreA repression is alleviated and transcripts critical for iron acquisition, including HapX, become more abundant. Also consistent with the observed SreA and HapX transcript profiles, AcuM (Afu2g12330) transcript levels were reduced at 2 and 6 hours growth in hypoxia and slightly increased at 12 and 24 hours. AcuM, a transcriptional regulator of gluconeogenic genes, has recently been suggested to repress SreA and thereby induce HapX transcripts via transcriptional profiling experiments [75]. It is unclear, however, whether *acuM *transcript levels were affected by changes in glucose levels between early and late time points or hypoxia itself. Importantly, the transcript profile of all the iron acquisition genes does not exactly mimic the expected profile based on what is known about the SreA and HapX regulons. Thus, it seems apparent that additional regulators of iron acquisition and homeostasis are present in *A. fumigatus *and operative under hypoxia.

## Conclusions

In conclusion, we present the first comprehensive examination of the transcriptional and proteomic response to hypoxia adaptation in the human fungal pathogen *A. fumigatus*. Recent data suggest that overcoming or tolerating hypoxia may be a key component of the virulence arsenal of this important human pathogen [7,10,27]. Overall, similar to the pathogenic yeast *C. albicans *and *C. neoformans*, we observed that the hypoxia response in *A. fumigatus *is characterized by both positive and negative changes in transcript and protein levels. Major themes of the hypoxia response in *A. fumigatus *observed in our study include: transcriptional and proteomic decreases in the TCA cycle in contrast with previous observations in *A. nidulans *and *C. neoformans*, ribosome biogenesis and purine metabolism and a concomitant increase in the oxidative stress response, glycolysis and fermentation, cell wall biosynthesis, and iron metabolism. A major positive response seen in transcript levels of genes involved in the biosynthesis of ergosterol was a major observation of the *A. fumigatus *hypoxia response and is consistent with similar observations in other fungi. Thus, changes in sterol levels in response to hypoxia are a major cellular response to oxygen limitation in the fungal Kingdom. A substantial number of transcription factors were positively regulated in response to hypoxia, including the known regulator of ergosterol biosynthesis, SrbA, and this was consistent with a strong positive effect of hypoxia on overall transcript levels. However, we also observed a core set of genes whose transcript levels decreased while protein levels increased in response to hypoxia. Thus, regulation of cellular hypoxia responses is likely multifaceted and future studies examining the key cellular regulatory responses during adaptation to hypoxia in *A. fumigatus *are thus warranted. Importantly, our analysis here will allow further investigation of the link between hypoxia adaptation and *A. fumigatus *pathogenesis. While the strong hypoxia growth deficit and virulence attenuation of the *A. fumigatus *SrbA null mutant strongly suggests a key link between hypoxia, sterol biosynthesis, and fungal virulence, our studies here suggest several potential sterol independent pathways that may be critical for hypoxia adaptation and thus potentially fungal virulence.

## Methods

### Strain and culture conditions

*Aspergillus fumigatus *wild-type strains ATCC 46645 and CBS144.89 were used for proteomic and transcriptomic analysis of the hypoxic response, respectively. *A. fumigatus *was grown in a fermenter (Biostat B-DCU-5l, Braun, Melsungen, Germany) as a batch culture (starting culture volume: 3l) at 37°C with constant stirring (550 rpm). The dissolved molecular oxygen was measured with a pO_2 _electrode (InPro6800/12/320, Mettler Toledo, Steinbach, Germany) connected to a measuring amplifier in a range of 0 - 10% pO_2 _(8842698 Braun, Melsungen, Germany). This setting allowed detection of low oxygen concentrations of 0.2% O_2 _and above. Aerobic culture conditions were established (21% O_2_) by aerating with 0.65 l/min of air. After an initial aerobic growth phase (between 12.5 h and 14 h) the oxygen concentration was set to a low oxygen partial pressure (hypoxic conditions of 0.2% O_2_). The O_2 _concentration was kept constant by aeration of the medium with a mixture of nitrogen and air at constant rate (0.65 l/min). The influx of air was controlled manually. The fungus was cultivated in *Aspergillus *minimal medium (AMM) as described previously [76] with slight modifications. The medium contained 16.65 mM glucose as sole carbon and energy source. The fermenter was inoculated with conidia to give a final concentration of 0.67 × 10^6 ^conidia/ml. Samples were taken after 0 h (after 12.5 h or 14 h aerobic growth), 2 h (for transcriptome analysis) or 3 h (for proteome analysis), 6 h, 12 h and 24 h cultivation at hypoxic growth conditions. In addition, the pH-value, glucose concentration (BIOSEN C-Line, EKF Diagnostic, Barleben, Germany) and dry weight biomass (HR73 Halogen Moisture Analyzer, Mettler Toledo, Steinbach, Germany) was determined. The concentrations of ethanol, D/L-lactate and acetate in the culture supernatant were quantified by enzymatic detection kits according to the manufacturer's instruction (UV-test for ethanol, D/L-lactate and acetate, R-Biopharm, Darmstadt, Germany). Harvested mycelium was filtered through miracloth (Merck KGaA, Darmstadt, Germany), rinsed with tap water and pressed to remove any liquid and immediately frozen in liquid nitrogen.

### Isolation of nucleic acids

Frozen mycelium was ground to a fine powder. 100 mg of tissue was used for total RNA isolation using the Ambion RNA isolation kit according to the manufacturer's instructions. The amount of RNA was determined spectrophotometrically with a nano-drop and RNA quality was interrogated with an Agilent Bioanalyzer.

### cDNA preparation and probe labelling

Ambion (Austin, TX) aRNA kit (AM1751) was used according to manufacturer's recommendations to amplify 1.5 ug of total RNA template. A final amount of 5 ug of aRNA was used for cDNA synthesis using SuperScriptIII (Invitrogen), following the "Microbial RNA aminoallyl labeling for microarrays" (SOP# M007 Rev. 2) protocol detailed at http://pfgrc.jcvi.org/index.php/microarray/protocols.html. Briefly, samples were RNaseH treated and concentration checked on Nanodrop. cDNA was purified with Qiagen QIAquick PCR purification kit. Samples were dried completely with speed-vac (Eppendorf). Pellet was suspended with 4.5 μL of 0.1 M Na_2_CO_3_. 4.5 uL of Cy3 or Cy5 dye was added to appropriate tubes, and incubated at 28°C for two hours. Uncoupled dye was removed with NaOAc-modified QIAquick PCR kit (Qiagen). Dye ratio was calculated with microarray analysis function on the Nanodrop-1000 (Thermo). The two differentially labelled probes (Cy3 vs. Cy5) that were hybridized to the same microarray slide are mixed with equal cDNA volumes. The Cy3/Cy5 probe mixture was dried to completion. Pellet was suspended in 10 μL of dH_2_O.

### Microarray hybridization

Spotted arrays (Aspergillus fumigatus Af293, version 3) from JCVI were used for the entire experiment http://pfgrc.jcvi.org/index.php/microarray/array_description/aspergillus_fumigatus/version3.html. The protocol "Microbial Hybridization of labelled probes" (SOP# M008 Rev 2.1) can be found at: http://pfgrc.jcvi.org/index.php/microarray/protocols.html. Briefly, the slides were soaked in sterile-filtered 5xSSC, 1%BSA, 0.2%SDS for two hours, washed and dried by centrifugation in mini slide spinner (LabNet) prior to hybridization. 45 μL of 50% formamide, 5xSSC, 0.1%SDS, 0.001 M DTT and 6 μL of salmon sperm DNA were added to previously rehydrated Cy3/Cy5 mix. Lifter slip (Erie Scientific) was washed in 100% EtOH and dried. The slide and lifter slip were placed in hybridization chamber (Corning) and 60 μL of probe mixture was pipeted under lifter slip. Chambers were sealed and incubated in 42°C water bath for 18 hours. Slides were washed twice in 2xSSC, 0.2% SDS, 0.02 M DTT, twice in 0.1× SSC, 0.1% SDS, 0.02 M DTT, twice in 0.1× SSC, 0.02 M DTT, and once with dH_2_O and 0.02 M DTT. Slides were dried completely in slide spinner and protected from UV exposure.

### Image processing

Slides were scanned with GenePix 4000 B dual wavelength scanner (Axon Instruments, Molecular Devices Co.), adjusting PMT gain ratio to ~1.0, 100% laser power, and pixel size of 10. The resulting images were checked by eye for misaligned regions or false signals using GenePixPro 6.0 (Axon Instruments, Molecular Devices Co.). A GenePix report file was generated with raw data reads for each spot.

### Data processing

All data are available at EMBL MIAMExpress (accession #E-MEXP-3251). Data were processed using TM4 software and protocol recommendations for microarray analysis http://www.tm4.org/. Briefly, GenePix files were converted to MeV files using Expressconverter 2.1. MeV files were analyzed with MIDAS 2.21 to normalize data, according to the recommended settings from TM4. Flip-dye pairs were read into MIDAS using a generous setting for one bad channel, and A and B channel flag check selected. LOWESS was used to minimize effect of intensity dependent bias, with default settings. Standard deviation regularization was used to minimize the effect of slide printing errors, with Cy3 as the reference. Flip-dye pairs were then checked for consistency and merged into a single MeV file. Biological replicates were then assigned a single median value for each gene and time point. Pathway analysis was then completed using gene set enrichment analysis GSEA. Briefly, the functional categories (metabolic pathways, protein families, protein domains) each gene belongs to were retrieved from the DAVID database [77]. For each time point genes were sorted by decreasing fold change. The method described previously [78], for which an implementation is available http://github.com/ajmazurie/xstats.enrichment, was then used to evaluate how enriched the top of each list (i.e., the most perturbed genes at each time point) was in any of the functional categories listed. The resulting p-values were then corrected for multiple testing using the FDR method [79]. SAM analysis was conducted in MeV, setting FDR at 0.05%. Functional category analysis was completed at the FungiFun website https://sbi.hki-jena.de/FungiFun/FungiFun.cgi to identify KEGG, GO and FunCat associated pathways for genes identified in the SAM included in the additional files [80]. Self Organizing Tree Algorithm (SOTA) analysis was completed in MeV with default settings to determine clusters. Self Organizing Map Algorithm (SOMA) was completed using the Cluster 1.5 library [81]. Self-organizing maps (also called Kohonen maps, see [82]) organize items into clusters on a two-dimensional grid in which two adjacent clusters are more similar than two distant clusters. As for other clustering methods such as K-means, self-organizing maps must be provided with the number of clusters to group the items into. However the spatial organization of these clusters allows for a visual validation of the number of clusters. Too many clusters will result in the centroid of neighbouring clusters to be nearly indistinguishable. As such the expression data were clustered with a grid of size 10 × 10 (100 clusters) down to 3x3 (9 clusters) using the Pearson correlation coefficient as the metric between expression profiles. The self-organizing map maximizing the number of clusters while limiting redundancies was the one of size 8 × 8 (64 clusters).

### Real-time RT-PCR

RNA from the microarray experiment was DNase treated with DNA-free kit (Ambion) and reverse transcribed with QuantiTect reverse transcription kit (Qiagen, USA). Primers for all genes of interest were designed with PrimerQuest (IDT) and manufactured by IDT, USA and sequences are listed in Additional file 8. All reactions were performed on BioRad MyIQ real-time PCR detection system with IQ SYBR green supermix (Bio-Rad, Hercules, CA). The ΔΔC_t _method was used to combine all datasets, using β-tubulin as the housekeeping gene [83].

### Sample Preparation for 2-D Gel Electrophoresis

Mycelial protein of *A. fumigatus *were cleaned up by trichloroacetic acid (TCA)/acetone precipitation as described previously, with slight modifications [84]. Frozen mycelium was ground in a precooled mortar in the presence of liquid nitrogen. About 100 mg of homogenate were precipitated over night with 300 μl 13.3% (w/v) TCA/0.3% (w/v) dithiothreitol (DTT)/acetone at -20°C. After centrifugation for 15 min at 12,000 × *g *at 4°C the supernatant was removed and the pellet was rinsed twice in ice-cold acetone containing 0.3% (w/v) DTT. The suspension was centrifuged again and the pellet was air-dried for 15 min at room temperature and subsequently resuspended in 300 μl 2D-lysis buffer (7 M urea, 2 M thiourea, 2% [w/v] CHAPS(3-[(3- cholamidopropyl)-dimethylammonio]-1- propanesulfonate), 1% [w/v] Zwittergent 3-10), 30 mM Tris). To improve protein solubility the samples were sonicated for 10 min in an ultrasonic bath and incubated for 1 h at -70°C. After centrifugation at 20,000 × *g *for 30 min at 16°C, the supernatant was collected. The pH of the samples was adjusted to 8.5 by the addition of a few microliters of a 100 mM NaOH stock solution. The protein concentration was determined according to the Bradford method [85] using the BIO-RAD protein assay (BIORAD Lab., Hertfordshire, U.K.).

### 2-D Gel Electrophoresis Analysis

The DIGE (difference in gel electrophoresis) technique was used to analyze cytosolic protein samples of *A. fumigatus *cultivated under normoxic and hypoxic conditions and carried out as described previously [86]. 15 Samples from three independent hypoxic cultivations were labeled with CyDye minimal dyes according to the manufacturer's protocol with slight modifications (GE Healthcare Bio-Sciences, Munich Germany). 50 μg of protein of each sample were labeled with 300 pmol of CyDye DIGE flourophores (dissolved in dimethyl formamide). Samples obtained at different time points 0, 3, 6, 12 and 24 h of hypoxic (0.2% pO_2_) conditions were labeled either with Cy3 or Cy5. A pool of all 15 samples (5 time points of 3 biological replicates) was prepared, labeled with Cy2, and used as a global internal standard. Samples were mixed and incubated for 30 min in the dark on ice. The reaction was stopped by adding 1 μL of 10 mM L-lysine. An equal volume of 4× sample buffer (composition described above for the lysis buffer, plus 3.2% [v/v] SERVALYT ampholytes [SERVA Electrophoresis, Heidelberg, Germany] and 40 mM DTT) was added.

For the separation of proteins in the first dimension 24 cm IPG strips with a nonlinear pH range from both pH 3 to 7 and pH 7 to 11 (GE Healthcare Bio- Sciences) which had been rehydrated overnight (7 M urea, 2 M thiourea, 2% [w/v] CHAPS, 1% [w/v] Zwittergent 3_10, 0.002% [w/v] bromophenol blue, 0.5% [v/v] IPG buffer, 1.2% [v/v] De-Streak reagent [GE Healthcare Bio-Sciences]) were used as described [31]. Equal amounts of protein samples from two time points and the internal standard preparations were combined and mixed with 100 μg unlabeled protein extract of the samples (to increase the protein amount for subsequent mass spectrometry analysis) and applied via anodic cup loading to IPG strips. Isoelectric focusing of 24 cm strips was carried out according to the following protocol: 4 h at 300 V (gradient), 4 h at 600 V (gradient), 4 h at 1,000 V (gradient), 5 h at 8,000 V (gradient) and 48,000 V h at 8,000 V (step).

After isoelectric focusing the IPG strips were equilibrated for 15 min in 10 mL of equilibration buffer (6 M urea, 30% [v/v] glycerol, 2% [w/v] SDS (sodium dodecyl sulfate), 75 mM Tris, 0.002% [w/v] bromophenol blue) containing 1% (w/v) DTT and subsequently for 15 min in 10 mL of equilibration buffer containing 2.5% (w/v) iodoacetamide. For the separation of proteins in the second dimension, the Ettan DALT System (GE Healthcare Bio-Sciences) was used. SDS polyacrylamide gels (11-16% [w/v]) of 1.0 mm thickness were casted with the a 2DEoptimizer (Biometra, Göttingen, Germany). Separation conditions were as follows: 1 W/gel for 1 h followed by 15 W/gel for 4 h. Proteins were visualized by analyzing the gels with a Typhoon 9410 scanner (GE Healthcare Bio-Sciences) using a resolution of 100 μm.

Spot detection of cropped images was performed with the DeCyder software package (version 7.0). The following parameters were applied: detection sensitivity, estimated number of spots: 2000; Process exclude filter set: slope > 1.6 and volume < 10 000. Changes in the abundance of protein spots were regarded as significant with a threshold of 2-fold standard deviation difference. Gels of three independent experiments (each 5 time points, technical duplicates) were analyzed with the BVA software, and average ratios as well as t-test values for difference in protein expression were calculated for each spot. Only spots with a t-test value of below 0.05 were regarded as significantly regulated. In order to identify the differently expressed proteins by mass spectrometry (MS), the gels were post-stained with colloidal Coomassie Brilliant Blue according to published protocol [31] and protein spots were excised manually.

Protein spots were tryptically digested according to published protocol [87] with slight modifications. Extracted peptides were measured and identified on an Ultraflex I and Ultraflextreme MALDI-TOF/TOF device using flexControl 3.3 for data collection and flexAnalysis 3.3 spectra analysis/peak list generation (Bruker Daltonics, Germany) as described previously [88]. Peptide mass fingerprint (PMF) and peptide fragmentation fingerprint (PFF) spectra were submitted to the MASCOT server (MASCOT 2.3, Matrix Science, U.K.), searching the NCBInr (monthly update) database limited to the taxon Fungi. With respect to the sample preparation, fixed modification of cysteine thiols to S-carbamidomethyl derivatives and variable methionine oxidation were defined for the database search. Further, up to one missed cleavage, and a peptide mass tolerance of 100 ppm was allowed. Results were regarded as significant with an allowed likelihood for a random hit of p ≤ 0.05, according to the MASCOT score (> 54). All proteome data (gel images, spot information) including mzML data files were imported into our in-house data warehouse Omnifung http://www.omnifung.hki-jena.de and are publicly accessible [89]. Identified proteins were classified with the FungiFun annotation tool [80].

## Authors' contributions

OK and RAC designed research. BMB, KK, MV, OK and RAC performed research. AM contributed analytical tools. AM, KK, MV, BMB, OK and RAC analyzed data. BMB, OK and RAC wrote the paper. All authors read and approved the final manuscript.

## Supplementary Material

Additional file 1 **Significance Analysis of Microarray (SAM) clusters of microarray data**. Microarray heat maps compare wild type ***Aspergillus fumigatus ***transcript levels at the indicated times after exposure to hypoxic conditions to the time point immediately prior to hypoxia exposure (0 hours) for significantly altered expression patterns as determined by SAM with a false discovery rate of 0.05. Each microarray slide pair was dye-swapped and three biological replicates were completed. Each gene was represented in duplicate on the slide array. The median expression value was retained for each gene among the technical and biological replicates. Yellow indicates an increase in expression, blue is a decrease. Click here for file

Additional file 2 **GSEA analysis of all microarray data**. Excel file of all genes in microarray experiment for all time points. Functional categories in column one include KEGG pathway, COG ontology, PIR keyword, sequence features, COG name, BLOCKS domain, INTERPRO, PRODOM protein family, PFAM domain, TIGRFAMS protein family, PROSITE domain, PIR superfamily, SMART domain, PRINT domain, PANTHER family and sub-family. Categories that are more significantly down are coded in blue, those that are up are coded in yellow. For those that have url links, these are included in the second column. Scores are separated into time points, either up or down regulated in the final 8 columns. Click here for file

Additional file 3 **Protein identification**. Word file with each labelled spot from Figure 2 identified. Average ratios compared between hypoxic (3, 6, 12, 24 hours) and normoxic conditions (0 hour). Statistical analyses of DIGE gels were performed by Decyder 7.0. Click here for file

Additional file 4 **Fold changes for both proteomic and transcriptomic data**. Excel file with all fold change values used to analyse the correlation between protein and mRNA abundance data. Genes with variable transcript abundance patterns are highlighted. Click here for file

Additional file 5 **Fold changes with associated pathways**. Excel file with median log2 expression values from microarray experiments used to generate heat maps. The first tab in the file contains the expression data for every transcript on the microarray. Additional functional categories are included that do not have heat maps in figures or additional files, and these are labelled on each tab in the excel spreadsheet. Click here for file

Additional file 6 **Functional categories of significantly increased transcripts**. Word file of pathway analysis performed with FungiFun https://sbi.hki-jena.de/FungiFun/FungiFun.cgi showing significant categories of increased transcripts. FungiFun is a web server that assigns functional annotations to fungal genes or proteins. Based on different classification methods like FunCat (Functional Catalogue), GO (Gene Ontology) and KEGG (Kyoto Encyclopedia of Genes and Genomes), FungiFun categorizes genes and proteins for fungal species on different levels and conducts an enrichment analysis. Click here for file

Additional file 7 **Functional categories of significantly decreased transcripts**. Word file of pathway analysis performed with FungiFun https://sbi.hki-jena.de/FungiFun/FungiFun.cgi showing significant categories of reduced transcripts. FungiFun is a web server that assigns functional annotations to fungal genes or proteins. Based on different classification methods FunCat, GO and KEGG, this program categorizes genes and proteins for fungal species on different levels and conducts an enrichment analysis. Click here for file

Additional file 8 **Primers**. Sequences of real time RT-PCR primers used in the present study. Click here for file
