# Supplementary figures and images for: Transcriptomic and proteomic analyses of the Aspergillus fumigatus hypoxia response using an oxygen-controlled fermenter
Source: BMC Genomics. 2012 Feb 6;13:62. doi: 10.1186/1471-2164-13-62 (PMC3293747; doi:10.1186/1471-2164-13-62)

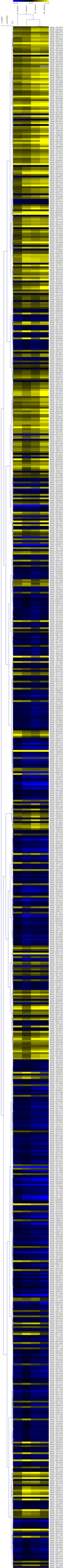

Supplement: Additional file 1 — Significance Analysis of Microarray (SAM) clusters of microarray data. Microarray heat maps compare wild type Aspergillus fumigatus transcript levels at the indicated times after exposure to hypoxic conditions to the time point immediately prior to hypoxia exposure (0 hours) for significantly altered expression patterns as determined by SAM with a false discovery rate of 0.05. Each microarray slide pair was dye-swapped and three biological replicates were completed. Each gene was represented in duplicate on the slide array. The median expression value was retained for each gene among the technical and biological replicates. Yellow indicates an increase in expression, blue is a decrease. [file 1471-2164-13-62-S1.TIFF]
