# Supplementary material for: Transcriptomic and proteomic analyses of the Aspergillus fumigatus hypoxia response using an oxygen-controlled fermenter
Source: BMC Genomics. 2012 Feb 6;13:62. doi: 10.1186/1471-2164-13-62 (PMC3293747; doi:10.1186/1471-2164-13-62)
Supplement: Additional file 3 — Protein identification. Word file with each labelled spot from Figure 2 identified. Average ratios compared between hypoxic (3, 6, 12, 24 hours) and normoxic conditions (0 hour). Statistical analyses of DIGE gels were performed by Decyder 7.0. [file 1471-2164-13-62-S3.DOCX]

# Additional file 3. *Aspergillus fumigatus* Proteomic Response to Short Term Hypoxia

| **Spot no^a)^** | **Locus tag** | **Putative function and protein name** | **3h^b)^** | **6h^b)^** | **12h^b)^** | **24h^b)^** | **pI^c)^** | **MW^c)^** |  |  |  |  |  |  |
| --- | --- | --- | --- | --- | --- | --- | --- | --- | --- | --- | --- | --- | --- | --- |
|  |  |  |  |  |  |  |  | **[kDa]** |  |  |  |  |  |  |
|  | **Glycolysis/Pyruvate metabolism** | | | | | | | |  |  |  |  |  |  |
| **1** | AFUA_5G01030 | Glyceraldehyde 3-phosphate dehydrogenase | -0.69 | -0.08 | 1.83 | 2.58 | 6.2 | 36.1 |  |  |  |  |  |  |
| **2** | AFUA_1G10350 | Phosphoglycerate kinase PgkA | 0.35 | 0.52 | 1.78 | 2.59 | 6.3 | 44.8 |  |  |  |  |  |  |
| **3** | AFUA_5G01970 | Glyceraldehyde 3-phosphate dehydrogenase GpdA | 0.51 | 0.73 | 1.02 | 1.92 | 7.0 | 36.3 |  |  |  |  |  |  |
| **4** | AFUA_5G13450 | Triosephosphate isomerase | 0.25 | 1.07 | 1.82 | 1.68 | 5.9 | 28.1 |  |  |  |  |  |  |
| **5** | AFUA_2G09790 | Glucose-6-phosphate isomerase | -1.40 | -1.99 | -2.25 | -2.47 | 5.9 | 61.3 |  |  |  |  |  |  |
| **6** | AFUA_1G06960 | Pyruvate dehydrogenase E1 component alpha subunit | 1.79 | 2.88 | 4.48 | 2.79 | 6.4 | 41.5 |  |  |  |  |  |  |
| **7** | AFUA_6G06770 | Enolase/allergen Asp F 22 | 0.22 | 0.68 | 0.31 | 0.24 | 5.4 | 47.3 |  |  |  |  |  |  |
| **8** | AFUA_3G11690 | Fructose-bisphosphate aldolase, class II | -1.85 | -2.40 | -1.96 | -0.23 | 5.6 | 39.8 |  |  |  |  |  |  |
| **9** | AFUA_3G09290 | Phosphoglycerate mutase, 2,3-bisphosphoglycerate-independent | 0.20 | 0.16 | -1.24 | -1.84 | 5.7 | 92.4 |  |  |  |  |  |  |
|  |  |  |  |  |  |  |  |  |  |  |  |  |  |  |
|  | **Pentose phosphate pathway** | | | | | | | |  |  |  |  |  |  |
| **10** | AFUA_6G08050 | 6-phosphogluconate dehydrogenase Gnd1 | -1.26 | -1.35 | -1.83 | -2.24 | 5.9 | 55.8 |  |  |  |  |  |  |
| **11** | AFUA_1G13500 | Transketolase TktA | -0.50 | -1.20 | -2.42 | -2.03 | 6.1 | 74.8 |  |  |  |  |  |  |
|  |  |  |  |  |  |  |  |  |  |  |  |  |  |  |
|  | **Amino acid metabolism** | | | | | | | |  |  |  |  |  |  |
|  | Aspartate |  |  |  |  |  |  |  |  |  |  |  |  |  |
| **12** | AFUA_4G10410 | Aspartate aminotransferase | 0.41 | 1.58 | 2.01 | 1.34 | 8.9 | 47.9 |  |  |  |  |  |  |
|  | Glutamate |  |  |  |  |  |  |  |  |  |  |  |  |  |
| **13** | AFUA_4G06620 | Glutamate/Leucine/Phenylalanine/Valine dehydrogenase | -0.21 | -0.26 | -0.07 | 1.44 | 5.8 | 49.4 |  |  |  |  |  |  |
| **14** | AFUA_4G13120 | Glutamine synthetase | -0.16 | 0.31 | 1.04 | 1.51 | 5.5 | 39.9 |  |  |  |  |  |  |
|  | Lysine |  |  |  |  |  |  |  |  |  |  |  |  |  |
| **15** | AFUA_6G04800 | Lysine decarboxylase-like protein | 0.30 | 0.64 | 2.17 | 3.32 | 9.2 | 30.8 |  |  |  |  |  |  |
| **16** | AFUA_3G11710 | Saccharopine dehydrogenase Lys1 | 0.21 | 0.42 | 2.70 | 2.73 | 5.2 | 41.2 |  |  |  |  |  |  |
|  | Methionine |  |  |  |  |  |  |  |  |  |  |  |  |  |
| **17** | AFUA_4G07360 | Cobalamin-independent methionine synthase MetH/D | -1.89 | -2.54 | -3.08 | -3.36 | 6.3 | 86.9 |  |  |  |  |  |  |
| **18** | AFUA_1G10630 | S-adenosylmethionine synthetase | -0.70 | -0.88 | -1.74 | -1.88 | 5.7 | 42.2 |  |  |  |  |  |  |
|  | Valine/Leucine/isoleucine | | | | | | | |  |  |  |  |  |  |
| **19** | AFUA_4G07210 | Mitochondrial acetolactate synthase small subunit | -0.04 | 0.10 | 1.91 | 2.10 | 6.4 | 35.6 |  |  |  |  |  |  |
| **20** | AFUA_1G15780 | 3-isopropylmalate dehydrogenase Leu2A | -0.19 | -1.00 | -2.11 | -2.07 | 5.3 | 39.0 |  |  |  |  |  |  |
| **21** | AFUA_3G14490 | Ketol-acid reductoisomerase | 0.40 | -0.44 | -1.07 | -1.66 | 9.3 | 56.4 |  |  |  |  |  |  |
|  | Cysteine |  |  |  |  |  |  |  |  |  |  |  |  |  |
| **22** | AFUA_1G10130 | Adenosylhomocysteinase | 0.48 | 0.14 | -1.12 | -1.33 | 5.8 | 48.5 |  |  |  |  |  |  |
|  | Histidine |  |  |  |  |  |  |  |  |  |  |  |  |  |
| **23** | AFUA_2G06230 | Glutamine amidotransferase:cyclase | 0.81 | 1.20 | 1.89 | 1.74 | 5.5 | 60.2 |  |  |  |  |  |  |
|  | Arginine and proline | | | | | | | |  |  |  |  |  |  |
| **24** | AFUA_6G08750 | Delta-1-pyrroline-5-carboxylate dehydrogenase PrnC | 0.17 | 0.94 | 2.00 | 3.04 | 8.3 | 63.0 |  |  |  |  |  |  |
|  |  |  |  |  |  |  |  |  |  |  |  |  |  |  |
|  | **Translation initiation, translation and protein biosynthesis** | | | | | | | |  |  |  |  |  |  |
| **25** | AFUA_2G09870 | Eukaryotic translation initiation factor 3 subunit EifCg, putative | 2.61 | 2.12 | 1.26 | 0.91 | 9.0 | 31.9 |  |  |  |  |  |  |
| **26** | AFUA_1G12170 | Translation elongation factor EF-Tu | 2.10 | 3.26 | 3.79 | 4.76 | 6.7 | 48.3 |  |  |  |  |  |  |
|  |  |  |  |  |  |  |  |  |  |  |  |  |  |  |
|  | **Secondary metabolism** | | | | | | | |  |  |  |  |  |  |
| **27** | AFUA_8G00550 | Methyltransferase SirN-like Pseurotin A Cluster | 2.83 | 4.68 | 7.81 | 8.57 | 5.2 | 29.6 |  |  |  |  |  |  |
| **28** | AFUA_4G04380 | Phenazine biosynthesis-like protein | 0.00 | 1.29 | 3.15 | 4.17 | 5.9 | 34.8 |  |  |  |  |  |  |
| **29** | AFUA_6G12220 | Isochorismatase family hydrolase | 5.02 | 6.16 | 7.33 | 8.03 | 5.9 | 20.9 |  |  |  |  |  |  |
|  |  |  |  |  |  |  |  |  |  |  |  |  |  |  |
|  | **TCA cycle** |  |  |  |  |  |  |  |  |  |  |  |  |  |
| **30** | AFUA_3G07810 | Succinate dehydrogenase subunit Sdh1 | 0.92 | -0.08 | -0.66 | -0.60 | 6.5 | 71.1 |  |  |  |  |  |  |
| **31** | AFUA_5G04230 | Citrate synthase (Cit1) | -0.11 | 0.55 | -0.95 | -0.62 | 8.7 | 52.1 |  |  |  |  |  |  |
| **32** | AFUA_7G05740 | Malate dehydrogenase, NAD-dependent | -1.45 | -1.84 | -2.31 | -1.91 | 9.1 | 35.9 |  |  |  |  |  |  |
| **33** | AFUA_6G06370 | NAD(+)-isocitrate dehydrogenase subunit I | 1.60 | 1.34 | 1.69 | 1.71 | 8.4 | 49.7 |  |  |  |  |  |  |
| **34** | AFUA_6G12930 | Mitochondrial aconitate hydratase, putative | 0.25 | -0.84 | -0.46 | -1.81 | 6.3 | 85.5 |  |  |  |  |  |  |
| **35** | AFUA_6G02470 | Fumarate hydratase | 0.24 | 0.05 | 1.92 | 1.14 | 9.1 | 63.2 |  |  |  |  |  |  |
|  |  |  |  |  |  |  |  |  |  |  |  |  |  |  |
|  | **Vitamine and cofactor biosynthesis** | | | | | | | |  |  |  |  |  |  |
| **36** | AFUA_5G02470 | Thiamine biosynthesis protein (Nmt1) | -1.05 | -1.09 | 0.74 | 3.00 | 6.0 | 38.3 |  |  |  |  |  |  |
| **37** | AFUA_8G04650 | 3-hydroxyanthranilate 3,4-dioxygenase Bna1 | 1.50 | 3.33 | 4.26 | 4.36 | 5.6 | 21.7 |  |  |  |  |  |  |
|  |  |  |  |  |  |  |  |  |  |  |  |  |  |  |
|  | **Electron transport and oxidative phosphorylation** | | | | | | | |  |  |  |  |  |  |
| **38** | AFUA_2G03010 | Cytochrome c subunit Vb | 0.41 | 1.21 | 2.83 | 3.55 | 6.1 | 21.5 |  |  |  |  |  |  |
| **39** | AFUA_1G03510 | ATP synthase gamma chain, mitochondrial precursor | 1.21 | 0.36 | -0.11 | 1.24 | 8.3 | 32.4 |  |  |  |  |  |  |
|  |  |  |  |  |  |  |  |  |  |  |  |  |  |  |
|  | **Fatty acid metabolism** | | | | | | | |  |  |  |  |  |  |
| **40** | AFUA_4G11080 | Acetyl-coenzyme A synthetase FacA | -1.59 | -3.12 | -3.97 | -4.61 | 6.0 | 74.5 |  |  |  |  |  |  |
| **41** | AFUA_8G04000 | Acetyl-CoA acetyltransferase | 0.34 | 0.14 | 1.45 | 1.40 | 6.4 | 40.9 |  |  |  |  |  |  |
| **42** | AFUA_5G06500 | Acyl-CoA dehydrogenase family protein | 1.27 | 2.13 | 2.57 | 2.50 | 9.1 | 51.6 |  |  |  |  |  |  |
|  |  |  |  |  |  |  |  |  |  |  |  |  |  |  |
|  | **Sulfate assimilation** | | | | | | | |  |  |  |  |  |  |
| **43** | AFUA_6G09070 | 3'(2'),5'-bisphosphate nucleotidase | -1.94 | -2.06 | -2.80 | -2.01 | 5.6 | 44.2 |  |  |  |  |  |  |
|  |  |  |  |  |  |  |  |  |  |  |  |  |  |  |
|  | **Protein folding** |  |  |  |  |  |  |  |  |  |  |  |  |  |
| **44** | AFUA_2G13040 | Mitochondrial co-chaperone GrpE | -0.04 | 0.92 | 2.16 | 2.28 | 7.9 | 28.4 |  |  |  |  |  |  |
| **45** | AFUA_3G07430 | Peptidyl-prolyl cis-trans isomerase/cyclophilin | -1.70 | -1.36 | -1.76 | -1.46 | 7.8 | 17.7 |  |  |  |  |  |  |
|  |  |  |  |  |  |  |  |  |  |  |  |  |  |  |
|  | **Ribosome biogenesis** | | | | | | | |  |  |  |  |  |  |
| **46** | AFUA_1G15730 | 40S ribosomal protein S22 | -0.80 | -1.91 | -1.98 | -2.66 | 9.7 | 14.7 |  |  |  |  |  |  |
| **47** | AFUA_2G03590 | 40S ribosomal protein S21 | -1.44 | -2.58 | -2.75 | -2.80 | 8.5 | 10.0 |  |  |  |  |  |  |
| **48** | AFUA_1G03390 | 60S ribosomal protein L12 | -0.51 | -1.33 | -1.87 | -2.22 | 9.8 | 18.2 |  |  |  |  |  |  |
| **49** | AFUA_6G12660 | 40S ribosomal protein S10b | 2.89 | 2.77 | 1.07 | 1.55 | 9.7 | 17.9 |  |  |  |  |  |  |
| **50** | AFUA_4G13170 | G-protein complex beta subunit CpcB | 0.01 | 0.46 | -0.98 | -1.77 | 6.1 | 35.0 |  |  |  |  |  |  |
|  |  |  |  |  |  |  |  |  |  |  |  |  |  |  |
|  | **Oxidative stress response** | | | | | | | |  |  |  |  |  |  |
| **51** | AFUA_4G09110 | Cytochrome c peroxidase Ccp1 | 0.79 | 0.95 | 1.61 | 1.64 | 8.6 | 40.4 |  |  |  |  |  |  |
| **52** | AFUA_6G02280 | Allergen Asp F3 | 0.25 | 0.26 | 0.54 | 3.09 | 5.4 | 18.5 |  |  |  |  |  |  |
| **53** | AFUA_5G01440 | Allergen | -0.17 | 0.20 | 1.24 | 2.20 | 8.4 | 22.0 |  |  |  |  |  |  |
|  |  |  |  |  |  |  |  |  |  |  |  |  |  |  |
|  | **Purine metabolism** | | | | | | | |  |  |  |  |  |  |
| **54** | AFUA_4G07690 | Phosphoribosylaminoimidazolecarboxamide formyltransferase/IMP cyclohydrolase | -0.54 | -1.55 | -1.20 | -2.02 | 6.4 | 65.0 |  |  |  |  |  |  |
| **55** | AFUA_5G06390 | Adenosine kinase | 0.71 | 0.32 | -1.05 | -1.60 | 5.0 | 36.9 |  |  |  |  |  |  |
| **56** | AFUA_1G07530 | Adenylate kinase | 0.70 | 0.31 | -0.55 | -0.83 | 7.7 | 28.7 |  |  |  |  |  |  |
| **57** | AFUA_5G03490 | Nucleoside diphosphate kinase | -1.76 | -1.55 | -2.44 | -2.37 | 7.8 | 16.9 |  |  |  |  |  |  |
|  |  |  |  |  |  |  |  |  |  |  |  |  |  |  |
|  | **Cellular one-carbon pathways** | | | | | | | |  |  |  |  |  |  |
| **58** | AFUA_3G09320 | Serine hydroxymethyltransferase | -0.75 | -0.83 | -2.67 | -2.37 | 7.6 | 51.9 |  |  |  |  |  |  |
|  |  |  |  |  |  |  |  |  |  |  |  |  |  |  |
|  | **mRNA synthesis and processing** | | | | | | | |  |  |  |  |  |  |
| **59** | AFUA_4G07660 | ATP dependent RNA helicase (Dbp1) | -0.56 | -2.75 | -3.00 | -3.36 | 9.1 | 72.1 |  |  |  |  |  |  |
|  |  |  |  |  |  |  |  |  |  |  |  |  |  |  |
|  | **Nucleic acid binding** | | | | | | | |  |  |  |  |  |  |
| **60** | AFUA_6G13330 | RNA binding protein | 4.22 | 5.20 | 5.75 | 3.17 | 5.7 | 12.5 |  |  |  |  |  |  |
| **61** | AFUA_1G09810 | PUA RNA binding domain protein | -1.18 | -1.16 | -1.05 | 0.00 | 9.1 | 19.7 |  |  |  |  |  |  |
| **62** | AFUA_7G01480 | Cap binding protein | -0.36 | -0.97 | -0.43 | -1.06 | 5.6 | 30.1 |  |  |  |  |  |  |
| **63** | AFUA_1G14120 | Nuclear segregation protein (Bfr1) | 1.10 | 0.06 | -0.36 | -2.09 | 9.1 | 57.4 |  |  |  |  |  |  |
| **64** | AFUA_2G10030 | Actin cytoskeleton protein (VIP1) | 1.07 | 1.95 | 2.69 | 2.70 | 5.9 | 28.3 |  |  |  |  |  |  |
|  |  |  |  |  |  |  |  |  |  |  |  |  |  |  |
|  | **Stress response** |  |  |  |  |  |  |  |  |  |  |  |  |  |
| **65** | AFUA_3G00590 | Asp-hemolysin | 3.49 | 4.93 | 7.10 | 7.62 | 5.3 | 15.2 |  |  |  |  |  |  |
| **66** | AFUA_3G14540 | Heat shock protein Hsp30/Hsp42 | 4.86 | 5.71 | 5.78 | 3.73 | 6.1 | 20.5 |  |  |  |  |  |  |
| **67** | AFUA_4G03410 | Flavohemoprotein | 4.08 | 4.78 | 7.10 | 8.03 | 5.7 | 45.6 |  |  |  |  |  |  |
|  |  |  |  |  |  |  |  |  |  |  |  |  |  |  |
|  | **Protein processing (proteolytic)** | | | | | | | |  |  |  |  |  |  |
| **68** | AFUA_1G14200 | Mitochondrial processing peptidase beta subunit | 0.23 | 0.63 | 0.24 | 0.74 | 5.9 | 53.3 |  |  |  |  |  |  |
| **69** | AFUA_6G08310 | Proteasome component Pre2 | 1.46 | 1.60 | 2.29 | 2.30 | 6.3 | 32.7 |  |  |  |  |  |  |
|  |  |  |  |  |  |  |  |  |  |  |  |  |  |  |
|  | **Alcohol fermentation** | | | | | | | |  |  |  |  |  |  |
| **70** | AFUA_3G11070 | Pyruvate decarboxylase PdcA | 1.30 | 1.41 | 2.42 | 2.47 | 6.1 | 63.0 |  |  |  |  |  |  |
| **71** | AFUA_7G01010 | Alcohol dehydrogenase AlcA | 11.16 | 12.29 | 12.19 | 12.52 | 7.0 | 37.6 |  |  |  |  |  |  |
|  |  |  |  |  |  |  |  |  |  |  |  |  |  |  |
|  | **Unclassified and proteins of unknown function** | | | | | | | |  |  |  |  |  |  |
| **72** | AFUA_4G11250 | Carbonic anhydrase Nce103 | 0.55 | 1.45 | 1.71 | 2.82 | 8.6 | 30.8 |  |  |  |  |  |  |
| **73** | AFUA_3G01580 | GMC oxidoreductase | -0.23 | 0.02 | 1.72 | 1.67 | 7.1 | 72.2 |  |  |  |  |  |  |
| **74** | AFUA_7G05070 | FAD dependent oxidoreductase, putative | 1.53 | 2.67 | 4.17 | 4.76 | 5.7 | 51.3 |  |  |  |  |  |  |
| **75** | AFUA_5G02870 | Oxidoreductase, short-chain dehydrogenase/reductase family | -0.12 | -0.75 | -1.62 | -2.75 | 5.4 | 27.4 |  |  |  |  |  |  |
| **76** | AFUA_7G00350 | Conserved hypothetical protein | -2.43 | -2.81 | -2.96 | -1.96 | 9.2 | 49.6 |  |  |  |  |  |  |
| **77** | AFUA_6G10450 | Conserved hypothetical protein | 2.10 | 3.60 | 4.65 | 4.55 | 9.3 | 26.7 |  |  |  |  |  |  |
| **78** | AFUA_4G09810 | Hypothetical protein | 1.71 | 2.31 | 3.92 | 4.82 | 9.4 | 31.7 |  |  |  |  |  |  |
| **79** | AFUA_5G14680 | Conserved hypothetical protein | 4.12 | 5.37 | 5.38 | 5.04 | 4.8 | 25.4 |  |  |  |  |  |  |
| **80** | AFUA_1G15140 | Mitochondrial phosphate carrier protein (Mir1) | -0.66 | -1.25 | -2.18 | -3.33 | 9.4 | 33.8 |  |  |  |  |  |  |
| **81** | AFUA_2G15430 | Sorbitol/xylulose reductase Sou1-like | 0.59 | 1.85 | 2.94 | 3.78 | 6.0 | 28.2 |  |  |  |  |  |  |
| **82** | AFUA_1G09800 | GTP-binding protein YchF | 0.94 | 0.78 | 0.70 | -0.16 | 7.6 | 43.5 |  |  |  |  |  |  |
| **83** | AFUA_2G00570 | GNAT family acetyltransferase | 1.58 | 1.81 | 3.27 | 4.70 | 5.9 | 28.5 |  |  |  |  |  |  |
| **84** | AFUA_2G11010 | Dihydroorotate reductase PyrE | 0.24 | 0.95 | 1.10 | 1.07 | 9.1 | 56.8 |  |  |  |  |  |  |
| **85** | AFUA_6G14460 | Similar to haloalkanoic acid dehalogenase | 0.17 | 0.49 | 2.57 | 3.24 | 6.2 | 26.7 |  |  |  |  |  |  |
| **86** | AFUA_1G04620 | Alcohol dehydrogenase, zinc-containing | 0.94 | 0.94 | 1.63 | 2.01 | 6.2 | 37.9 |  |  |  |  |  |  |
| ^a)^ | Spot number in Figure 2. | | | | | | | |  |  |  |  |  |  |
| ^b)^ | Average ratios compared under hypoxic (3, 6, 12, 24 h) and normoxic conditions (0 h). Statistical analysis of DIGE gels were performed by the | | | | | | | | | | | | | |
|  | Decyder 7.0 software programs DIA and BVA. | | | | | | | |  |  |  |  |  |  |
| ^c)^ | Theoretical values. |  |  |  |  |  |  |  |  |  |  |  |  |  |
|  |  | | | | | | | |  |  |  |  |  |  |
|  |  | | | | | | | |  |  |  |  |  |  |
