# Supplementary material for: Transcriptomic and proteomic analyses of the Aspergillus fumigatus hypoxia response using an oxygen-controlled fermenter
Source: BMC Genomics. 2012 Feb 6;13:62. doi: 10.1186/1471-2164-13-62 (PMC3293747; doi:10.1186/1471-2164-13-62)
Supplement: Additional file 6 — Functional categories of significantly increased transcripts. Word file of pathway analysis performed with FungiFun https://sbi.hki-jena.de/FungiFun/FungiFun.cgi showing significant categories of increased transcripts. FungiFun is a web server that assigns functional annotations to fungal genes or proteins. Based on different classification methods like FunCat (Functional Catalogue), GO (Gene Ontology) and KEGG (Kyoto Encyclopedia of Genes and Genomes), FungiFun categorizes genes and proteins for fungal species on different levels and conducts an enrichment analysis. [file 1471-2164-13-62-S6.DOC]

## Additional file 6 - Significant functional GO (Molecular and biological function), KEGG, and FunCat categories of significantly increased transcripts using FungiFun webserver [81] (p<0.03)

## Molecular function

**GO number category name hits p-value**

GO:0000166 nucleotide binding 4 4.317e-07

GO:0003824 catalytic activity 67 0.00088

GO:0016491 oxidoreductase activity 53 0.00151

GO:0005524 ATP binding 13 0.00416

GO:0016301 kinase activity 2 0.00425

GO:0019825 oxygen binding 2 0.00575

GO:0050662 coenzyme binding 6 0.01121

GO:0005506 iron ion binding 13 0.01462

GO:0043565 sequence specific DNA binding 7 0.01788

GO:0004075 biotin carboxylase activity 2 0.01806

GO:0051287 NAD binding 5 0.02048

GO:0004722 protein serine/threonine 2 0.02629

phosphatase activity

GO:0016765 alkyl or aryl transferase activity 2 0.02629

## Biological process

**GO number category name hits p-value**

GO:0055114 oxidation-reduction process 53 0.00102

GO:0008152 metabolic process 74 0.00123

GO:0006412 translation 1 0.00329

GO:0016310 phosphorylation 2 0.00426

GO:0006633 fatty acid biosynthetic process 5 0.00429

GO:0015671 oxygen transport 2 0.00575

GO:0006950 response to stress 6 0.00611

GO:0009058 biosynthetic process 9 0.01186

## KEGG categories

**KEGG number category name hits p-value**

1.3.5 Steroid biosynthesis 7 5.36e-06

2 Genetic information processing 4 2.93e-05

1.3 Lipid metabolism 19 0.00018

1 Metabolism 59 0.00039

0.1.1 Metabolic pathways 42 0.02636

2.3 Folding, sorting and degradation 1 0.02656

## FunCat categories

**FunCatID category name hits p-value**

11 Transcription 21 4.31e-05

10 Cell cycle and DNA processing 19 5.71e-05

16 Protein with binding function or 82 0.00015

co-factor requirement

12 Protein synthesis 4 0.00023

14 Protein fate 30 0.00043

01 Metabolism 138 0.00299

32 Cell rescue, defence and virulence 62 0.01145

42 Biogenesis of cellular components 28 0.01402
