# Supplementary material for: Transcriptomic and proteomic analyses of the Aspergillus fumigatus hypoxia response using an oxygen-controlled fermenter
Source: BMC Genomics. 2012 Feb 6;13:62. doi: 10.1186/1471-2164-13-62 (PMC3293747; doi:10.1186/1471-2164-13-62)
Supplement: Additional file 7 — Functional categories of significantly decreased transcripts. Word file of pathway analysis performed with FungiFun https://sbi.hki-jena.de/FungiFun/FungiFun.cgi showing significant categories of reduced transcripts. FungiFun is a web server that assigns functional annotations to fungal genes or proteins. Based on different classification methods FunCat, GO and KEGG, this program categorizes genes and proteins for fungal species on different levels and conducts an enrichment analysis. [file 1471-2164-13-62-S7.DOC]

## Additional file 7 - Significant functional GO (Molecular and biological function), KEGG, and FunCat categories of significantly increased transcripts using FungiFun webserver [81] (p<0.03)

## Molecular function

**GO number category name hits p-value**

GO:0003735 structural constituent of ribosome 29 1.11e-07

GO:0003723 RNA binding 28 4.04e-06

GO:0003700 sequence specific DNA binding 7 7.12e-06

transcription factor activity

GO:0003743 translation initiation factor activity 17 9.24e-06

GO:0004775 succinate-CoA ligase activity 4 5.89e-05

GO:0003824 catalytic activity 60 0.00011

GO:0000166 nucleotide binding 73 0.00011

GO:0008270 zinc ion binding 33 0.00046

GO:0003676 nucleic acid binding 46 0.00119

GO:0000287 magnesium ion binding 11 0.00139

GO:0003755 peptidyl-prolyl cis-trans 7 0.00205

isomerase activity

GO:0016853 isomerase activity 16 0.00226

GO:0070403 NAD+ binding 4 0.00309

GO:0000175 3’-5’-exoribonuclease activity 3 0.00589

GO:0003878 ATP citrate synthase activity 2 0.00771

GO:0004535 poly(A)-specific ribonuclease 2 0.00771

activity

GO:0004489 methylenetetrahydrofolate 2 0.00771

reductase (NADPH) activity

GO:0003862 3-isopropylmalate dehydrogenase 2 0.00771

activity

GO:0008553 hydrogen-exporting ATPase 2 0.00771

activity, phosphorylative mechanism

GO:0016491 oxidoreductase activity 50 0.01001

GO:0016616 oxidoreductase activity, acting 7 0.01096

on the CH-OH group of donors,

NAD or NADP as acceptor

GO:0051287 NAD binding 8 0.01121

GO:0008565 protein transporter activity 6 0.01527 GO:0016798 hydrolase activity, acting on 7 0.01569

glycosyl bonds

GO:0003756 protein disulphide isomerase 2 0.02177

activity

GO:0004749 ribose phosphate diphosphokinase 2 0.02177

activity

## Biological process

**GO number category name hits p-value**

GO:0006412 translation 47 9.85e-12

GO:0006413 translational initiation 17 9.24e-06

GO:0008152 metabolic process 68 2.06e-05

GO:0042254 ribosome biogenesis 11 0.00037

GO:0006476 protein deacetylation 4 0.00077

GO:0003642 chromatin silencing 4 0.00077

GO:0006355 regulation of transcription, 16 0.00089

DNA-dependent

GO:0016192 vesicle-mediated transport 10 0.00189

GO:0006351 transcription, DNA-dependent 18 0.00205

GO:0006465 signal peptide processing 3 0.00252

GO:0006886 intracellular protein transport 11 0.00470

GO:0009098 leucine biosynthetic process 3 0.00589

GO:0006555 methionine metabolic process 2 0.00771

GO:0009156 ribonucleoside monophosphate 2 0.00771

biosynthetic process

GO:0044267 cellular protein metabolic process 4 0.00805

GO:0006364 rRNA processing 10 0.00897

GO:0006099 tricarboxylic acid cycle 5 0.00990

GO:0009082 branched chain family amino acid 4 0.01178

biosynthetic process

GO:0006457 protein folding 12 0.01246

GO:0055114 oxidation-reduction process 50 0.01412

GO:0006414 translational elongation 5 0.01677

GO:0009165 nucleotide biosynthetic process 3 0.01804

GO:0044249 cellular biosynthetic process 2 0.02177

GO:0009097 isoleucine biosynthetic process 2 0.02177

GO:0031167 rRNA methylation 2 0.02177

GO:0008299 isoprenoid biosynthetic process 4 0.02217

GO:0006520 cellular amino acid metabolic 6 0.02684

process

GO:0006974 response to DNA damage stimulus 7 0.02990

## KEGG categories

**KEGG number category name hits p-value**

2.2.1 Ribosome 24 1.21e-07

2 Genetic information processing 81 8.29e-07

2.2 Translation 28 8.59e-06

1 Metabolism 113 0.00150

2.3 Folding, sorting and degradation 30 0.00202

1.1.2 Citrate cycle (TCA cycle) 9 0.00310

1.7.1 N-Glycan biosynthesis 8 0.00915

1.9.1 Terpenoid backbone biosynthesis 5 0.01680

1.5 Amino acid metabolism 23 0.01795

2.3.7 RNA degradation 10 0.02026

## FunCat category

**FunCat ID category name hits p-value**

12 Protein synthesis 86 3.41e-11

16 Protein with binding function 345 8.78e-09

or cofactor requirement

14 Protein fate 173 1.14e-05

01 Metabolism 248 1.86e-05

11 Transcription 142 0.00063

10 Cell cycle and DNA processing 128 0.00705

34 Interaction with the environment 50 0.02716

32 Cell rescue, defence and virulence 99 0.02963
